# Up-to-Date Pathologic Classification and Molecular Characteristics of Intrahepatic Cholangiocarcinoma

**DOI:** 10.3389/fmed.2022.857140

**Published:** 2022-03-31

**Authors:** Taek Chung, Young Nyun Park

**Affiliations:** ^1^Department of Biomedical Systems Informatics, Yonsei University College of Medicine, Seoul, South Korea; ^2^Department of Pathology, Graduate School of Medical Science, Brain Korea 21 Project, Yonsei University College of Medicine, Seoul, South Korea

**Keywords:** intrahepatic cholangiocarcinoma, pathology, small duct, large duct, tumor microenvironment, genomics, transcriptomics

## Abstract

Intrahepatic cholangiocarcinoma (iCCA) is an aggressive primary liver malignancy with an increasing incidence worldwide. Recently, histopathologic classification of small duct type and large duct type iCCA has been introduced. Both these types of tumors exhibit differences in clinicopathological features, mutational profiles, and prognosis. Small duct type iCCA is composed of non-mucin-producing cuboidal cells, whereas large duct type iCCA is composed of mucin-producing columnar cells, reflecting different cells of origin. Large duct type iCCA shows more invasive growth and poorer prognosis than small duct type iCCA. The background liver of small duct type iCCA often shows chronic liver disease related to hepatitis B or C viral infection, or alcoholic or non-alcoholic fatty liver disease/steatohepatitis, in contrast to large duct type iCCA that is often related to hepatolithiasis and liver fluke infection. Cholangiolocarcinoma is a variant of small duct type iCCA composed of naïve-looking cuboidal cells forming cords or ductule-like structures, and shows better prognosis than the conventional small duct type. Fibrous tumor stroma, one of the characteristic features of iCCA, contains activated fibroblasts intermixed with innate and adaptive immune cells. The types of stroma (mature versus immature) are related to tumor behavior and prognosis. Low tumor-infiltrating lymphocyte density, *KRAS* alteration, and chromosomal instability are related to immune-suppressive tumor microenvironments with resistance to programmed death 1/ programmed death ligand 1 blockade. Data from recent large-scale exome analyses have revealed the heterogeneity in the molecular profiles of iCCA, showing that small duct type iCCA exhibit frequent *BAP1*, *IDH1/2* hotspot mutations and *FGFR2* fusion, in contrast to frequent mutations in *KRAS*, *TP53*, and *SMAD4* observed in large duct type iCCA. Multi-omics analyses have proposed several molecular classifications of iCCA, including inflammation class and proliferation class. The inflammation class is enriched in inflammatory signaling pathways and expression of cytokines, while the proliferation class has activated oncogenic growth signaling pathways. Diverse pathologic features of iCCA and its associated multi-omics characteristics are currently under active investigation, thereby providing insights into precision therapeutics for patients with iCCA. This review provides the latest knowledge on the histopathologic classification of iCCA and its associated molecular features, ranging from tumor microenvironment to genomic and transcriptomic research.

## Introduction

Cholangiocarcinomas (CCAs) include intrahepatic CCA (iCCA), perihilar CCA, and distal CCA (1). Anatomically, iCCA, often called as “peripheral CCA,” is defined as a tumor located in the periphery of the second-order bile ducts, ranging from segmental bile ducts to smaller branches of the intrahepatic biliary tree. Perihilar CCA, also known as Klatskin tumor, is defined as a tumor that arises at the junction where the right and left hepatic ducts meet, with the insertion site of the cystic duct as its distal limit. CCAs involving more of the distal area, such as the common bile duct, is defined as distal CCA. This review mainly focuses on iCCA.

Recent evaluation indicates that iCCA comprises approximately 10–15% of primary liver malignancies (2, 3), and its incidence worldwide has increased over the past decades (4). However, changes in the nomenclature, classification, and the disease coding system of CCA have hampered the accurate estimation of the incidence of iCCA (5). Countries with the highest incidence include South Korea (2.8 per 100,000 people/year), where *Clonorchis sinensis* infection was prevalent in the past, and Thailand (2.2 per 100,000 people/year), which still is an endemic area for infections due to *Opisthorchis viverrini* (4, 6, 7). In other countries where parasites are not endemic, the incidence of iCCA is low, usually below or around 1 per 100,000 people/year. Moreover, there are proposed risk factors such as choledochal cyst, primary sclerosing cholangitis, chronic B or C viral hepatitis, and non-alcoholic fatty liver disease caused by obesity or metabolic syndromes. However, a significant proportion of patients with iCCA have no known risk factors (4, 8). Since these patients rarely present symptoms in the early stage, they are often diagnosed with advanced disease with a dismal prognosis and 5-year overall survival rate of approximately 10% even in developed countries (9–11).

Intrahepatic cholangiocarcinoma is an epithelial neoplasm with biliary differentiation, and usually presents with abundant fibrous tumor stroma containing cancer-associated fibroblasts (CAFs), innate and adaptive immune cells, etc. Recently, iCCAs have been classified into two subtypes namely, small duct type and large duct type (3, 12). Furthermore, the molecular characteristics of iCCA are under active investigation owing to technological advances in nucleotide sequencing and the availability of massive data sources on cancer, such as The Cancer Genome Atlas (TCGA) and cBio Cancer Genomics Portal (13, 14). The combination of histopathological and multi-omics data have provided novel insights into understanding the molecular pathology and thereby, developing therapeutic options for iCCA.

This review aims to provide the latest knowledge on the histopathologic classification of iCCA based on the fifth edition of the World Health Organization (WHO) classification of digestive system tumors. In addition, we discuss the associated molecular features based on tumor microenvironment, genomic and transcriptomic research results presented so far.

## Pathological Features of Intrahepatic Cholangiocarcinoma

Intrahepatic cholangiocarcinoma is an adenocarcinoma arising in the intrahepatic biliary tree. Fibrous tumor stroma is one of the characteristic features of iCCA, and the fibrous stroma is various in amount and distribution. The tumor center is usually more fibrotic than the tumor periphery, showing proliferating tumor cells invading into the surrounding liver. Lymphovascular and perineural invasion are often detected even at an early stage.

### Gross Features of Intrahepatic Cholangiocarcinoma

**FIGURE 1:**
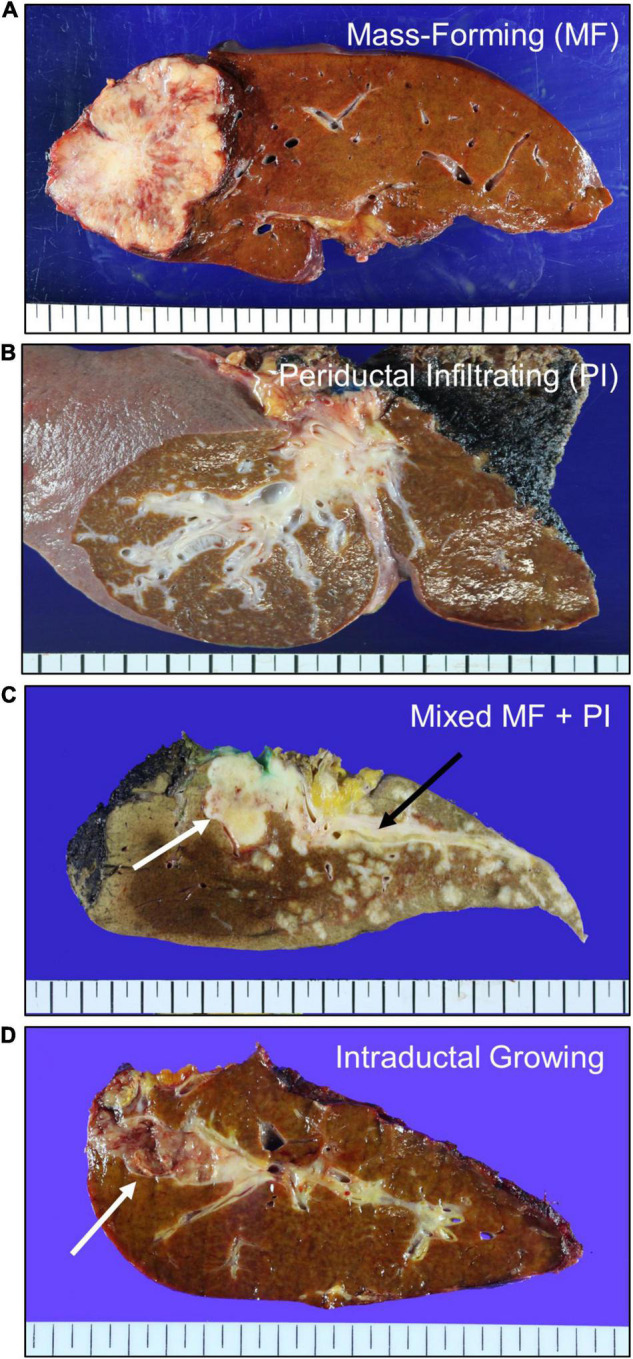
Macroscopic types of intrahepatic cholangiocarcinoma. **(A)** Mass-forming (MF) type showing a whitish tan tumor mass invading adjacent liver parenchyma. **(B)** Periductal infiltrating (PI) type showing a whitish periductal tumor growth along the bile duct branches. **(C)** Mixed type of MF and PI showing a solid tumor of MF type (white arrow) and a periductal growth of PI type (black arrow). **(D)** Intraductal growing type showing a friable tumor mass in the dilated bile duct (white arrow). This type of tumor is re-classified as intraductal papillary neoplasm of bile duct.

Macroscopically, iCCAs can be classified into three types: mass-forming, periductal infiltrating, and intraductal growing. Based on gross appearance, the mass-forming type is most common, and often exhibits mixed features (Figure 1) (15). The mass-forming type shows a definite round tumor mass with invasive border. The cut surface is usually white, pale tan or yellowish in color with firm consistency due to fibrous tumor stroma. The periductal infiltrating type shows a growth pattern that extends along the bile duct, exhibiting a whitish fibrotic and thickened bile duct wall. The intraductal growing type grows into the lumen of the bile duct, forming single or multiple soft papillary masses attached to the bile duct wall. The tumor mass and resultant obstruction often dilate the bile duct and make the tumor symptomatic. Since most of the intraductal growing type iCCA cases are now being considered as malignant transformations of intraductal papillary neoplasm of the bile duct (IPNB), in the latest fifth edition of WHO classification, gross morphologic types of iCCA include mass forming, periductal infiltrating and mixed type of these two (3, 16).

### Classification of Small Duct Type and Large Duct Type

**FIGURE 2:**
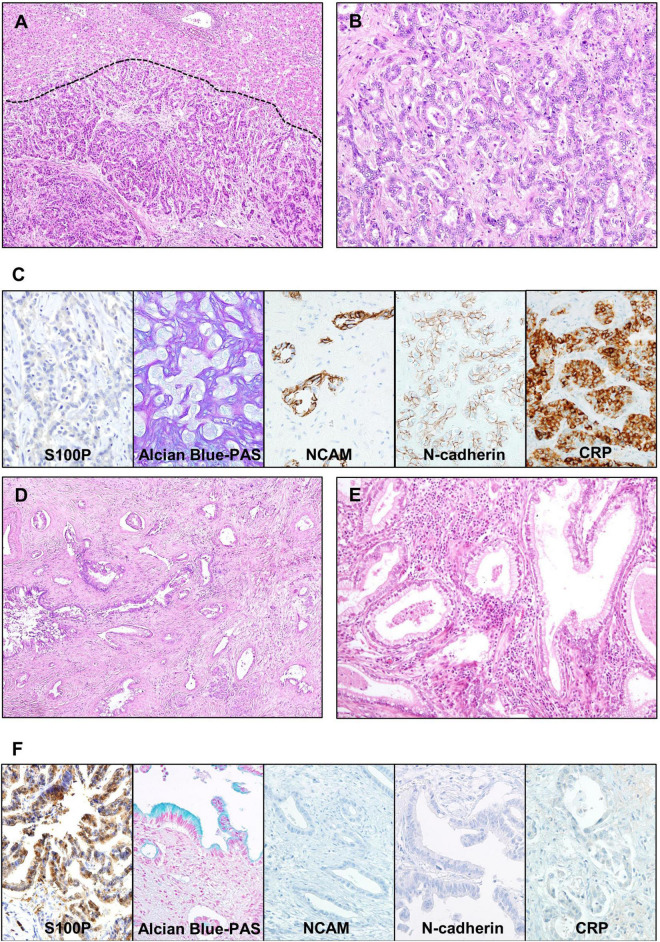
Representative microscopic images of small duct type and large duct type intrahepatic cholangiocarcinoma (iCCA). **(A–C)** Small duct type iCCA. **(A)** A low-power view showing uniform-shaped tumor glands replacing hepatocellular trabeculae at the border (indicated by dashed line). **(B)** A higher magnification image showing the growth of cuboidal cells forming cords and small glandular structures, without intra- or extracellular mucin. **(C)** Microscopic images of special and immunohistochemical panel staining for small duct type iCCA; positive expression of NCAM, N-cadherin and CRP, negative expression of S100P, and absence of mucin in the Alcian blue staining is characteristic. **(D–F)** Large duct type iCCA. **(D)** A low magnification image shows infiltrative growth of adenocarcinoma with rich fibrous stroma. **(E)** A higher magnification image showing columnar cells with intracellular mucin forming irregular glandular spaces. **(F)** Microscopic images of special and immunohistochemical panel staining for large duct type iCCA; positive expression of S100P, presence of mucin in the Alcian blue staining, and negative expression of NCAM, N-cadherin, and CRP is characteristic. Original magnification: 40× for **(A,D)**, 100× for **(B,E)**, 200× for **(C,F)**. S100P, S100 calcium-binding protein P; NCAM, neural cell adhesion molecule; CRP, c-reactive protein; PAS, periodic acid–Schiff.

Conventional iCCA can be further classified into two histopathological types according to the level or size of the affected duct. Recently, small duct type and large duct type iCCA have been introduced in the WHO classification (3). Small duct type iCCA, which has been reported as a peripheral, ductular, and cholangiolar type, accounts for 36–84% of iCCA (12, 17–19). Small duct type iCCA shows small-sized tubular growth of cuboidal or low-columnar tumor epithelial cells. There is little or no mucin production, and occasional areas of growth exhibit a pattern resembling ductular reaction with slit-like glandular lumen (Figures 2A,B). Background liver of small duct type iCCA often shows chronic liver disease related to B viral hepatitis, C viral hepatitis, alcoholic hepatitis, and non-alcoholic steatohepatitis (12, 17, 20).

Large duct type iCCA, which has been reported as bile duct type or perihilar type, arises in large intrahepatic bile ducts and comprises 8–60% of iCCA cases (12, 17–19). Large duct type iCCA is composed of mucin-producing columnar cells forming irregular shaped-and-sized tubules or gland-like structures. This type usually shows a highly invasive growth pattern accompanied by a desmoplastic reaction (Figures 2D,E) (3). Pathological examination of the background liver of large duct type iCCA often reveals chronic bile duct injury due to hepatolithiasis, parasitic infection in bile ducts, or primary sclerosing cholangitis (8, 20).

Furthermore, histopathological features of small duct type and large duct type iCCA are related to their gross appearance. The periductal infiltrative type of iCCA is exclusively large duct type, whereas the mass-forming type is more heterogeneous, including small duct and large duct types (17, 21). Mass-forming type iCCA with small duct type histology showed better prognosis than other types (17).

#### Putative Cells of Origin

Small duct type iCCA occurs in smaller intrahepatic bile ducts compared to large duct type. Canals of Hering, which are histological structures that link hepatic canaliculi and the biliary tree, cuboidal cholangiocytes of bile ductules, and interlobular bile ducts are considered as the putative cells of origin (22, 23). In contrast, large duct type iCCA might be derived from columnar biliary epithelium producing mucin or peribiliary glands around them (8, 20). However, the cellular origin of iCCA is still controversial, since various lineage tracing animal studies showed mixed results indicating hepatic stem or progenitor cells, cholangiocytes or hepatocytes as the cells of origin of iCCA (24, 25). Further research is required to conclusively define the origin of small duct and large duct type iCCAs.

#### Immunohistochemical Markers for Small Duct Type and Large Duct Type Intrahepatic Cholangiocarcinoma

Examination of a panel of immunohistochemical (IHC) markers is useful to differentiate small duct type and large duct type. In large duct type iCCAs, high expression of mucin (MUC) core protein 5AC, MUC6, and S100 calcium-binding protein P (S100P) has been reported (26, 27), whereas neural cell adhesion molecule (NCAM, also known as CD56) and N-cadherin have been found to be highly expressed in the small duct type. NCAM and N-cadherin are normally expressed in cholangioles (28, 29). Intra- and/or extracellular mucin, detected by mucicarmine or Alcian blue staining, is abundant in the large duct type in contrast to its scarcity or absence in the small duct type. Examining a panel of these markers, including S100P, N-cadherin, NCAM, and Alcian blue, has been reported to be more effective in differentiating small duct and large duct types (12). Additionally, c-reactive protein (CRP) was recently found to be an effective marker for the diagnosis of small duct type iCCA (30) (Figures 2C,F). Biliary cytokeratins (CKs) such as CK7 and CK19 are useful for confirming biliary differentiation or biliary origin. However, their ability to differentiate between small duct type and large duct type iCCA is limited (31, 32).

Histopathological features of hematoxylin and eosin-stained slides usually provide insight to distinguish small duct and large duct type iCCA. In addition, application of immunohistochemical (IHC) markers (S100P, NCAM, and N-cadherin) and special stain for mucin is useful to support the diagnosis of iCCA subtypes, especially when the tissue is limited in biopsies.

#### Comparison of Prognosis and Treatment Response Between Small Duct Type and Large Duct Type Intrahepatic Cholangiocarcinoma

The prognosis of small duct type iCCA is generally favorable compared to that of large duct type iCCA (12, 17, 33, 34). Accordingly, inflammation-related markers [CRP and fibrinopeptide B (FGB)] and proliferation-related markers [extracellular signal-regulated kinases (ERK) 1/2 and Ki-67] are highly expressed in small duct type iCCA and large duct type iCCA, respectively (17). It has also been reported that the response to conventional chemotherapy is better with small duct type than with large duct type iCCA (35).

#### Differential Diagnosis of Intrahepatic Cholangiocarcinoma in a Biopsied Tissue

For liver primary tumors, diagnosis of iCCA requires differentiation from combined hepatocellular-cholangiocarcinoma (cHCC-CC), since both components of cHCC-CC may not clearly present due to the limitations of the biopsied tissue. Application of IHC markers for hepatocellular carcinoma (HCC) is helpful to identify portions of hepatocytic differentiation. Hepatocyte paraffin-1 (Hep Par 1) and arginase-1 (ARG1) are highly sensitive and specific (both exceeding 80%) markers, and addition of glypican-3 (GPC) is shown to be useful for the diagnosis of poorly differentiated areas of hepatocytic differentiation with sensitivity over 80% (36).

Since an iCCA is histopathologically an adenocarcinoma, it is necessary to differentiate it from metastatic adenocarcinoma from other organs. Application of the following IHC markers is helpful. Caudal-type homeobox 2 (CDX2) is a widely used marker for the diagnosis of metastatic colorectal adenocarcinoma with sensitivity over 90%. Since its specificity is relatively low (70%) (37), combination with CK7 and CK20, which are usually negative and positive in colorectal adenocarcinoma, respectively, is recommended (32). Adenocarcinoma of the lung and ductal carcinoma of the breast, which can be differentiated by IHC staining with antibodies of thyroid transcription factor-1 (TTF-1; 75% sensitivity and specificity) (38, 39) and GATA binding protein 3 (GATA-3; over 90% sensitivity and specificity), respectively (38, 40, 41). Paired box 8 (PAX8) is a sensitive marker for ovarian and endometrial carcinomas, as well as for renal cell carcinomas with sensitivity approaching 90% (40). Metastatic prostate adenocarcinoma is usually positive for the antibodies against prostate specific antigen (PSA) and prostate specific acid phosphatase (PSAP), with sensitivity and specificity exceeding 95% (40, 42). However, in cases of metastatic adenocarcinomas originating from organs adjacent to the liver including gallbladder, pancreas, and stomach, etc., it is difficult to differentiate iCCA from these tumors, due to the lack of specific IHC marker. Some potentially promising markers have been introduced, and filamin A was reported to show high positivity (63%) by immunohistochemistry on iCCA (43). Recently, an *in situ* hybridization assay for albumin RNA was reported to show 90% sensitivity and 100% specificity for iCCA, particularly for the differentiation of small duct type iCCA and metastatic tumors (44–46).

### Variants of Intrahepatic Cholangiocarcinoma

#### Cholangiolocarcinoma

**FIGURE 3:**
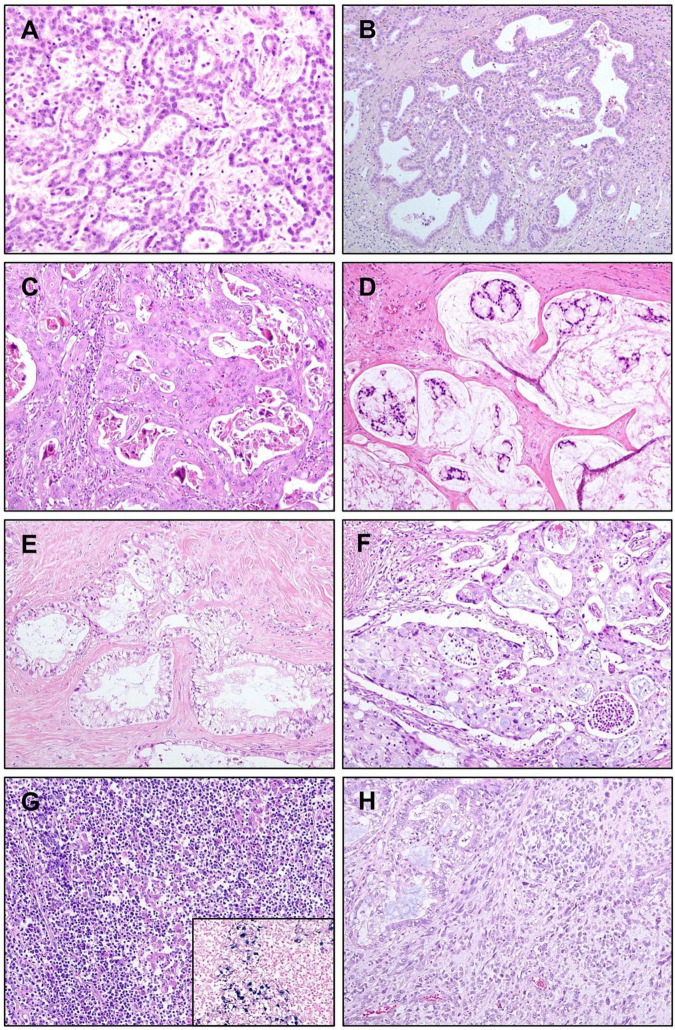
Variants of intrahepatic cholangiocarcinoma (iCCA). **(A)** Cholangiolocarcinoma. Bland-looking small cuboidal tumor epithelial cells are forming cords or ductules, with antler-like branching pattern. **(B)** iCCA with ductal plate malformation pattern. Cuboidal tumor cells are forming irregularly dilated and coalesced spaces, resembling developmental anomaly of ductal plate. **(C)** Adenosquamous carcinoma showing both of gland-forming portion and portions with squamous differentiation. **(D)** Mucinous carcinoma. Mucin-producing tumor cell clusters are floating in mucin pools. **(E)** Clear cell carcinoma. Tumor cells have large clear cytoplasm with eccentric nuclei. **(F)** Mucoepidermoid carcinoma showing squamoid tumor cells intermixed with mucin-producing cells. **(G)** Lymphoepithelioma-like carcinoma showing marked lymphocytic infiltration into tumor epithelial component. Tumor epithelial cells are positive for Epstein–Barr virus (EBV), detected by *in situ* hybridization of EBV-encoded small RNA (inset). **(H)** Sarcomatous iCCA showing mainly pleomorphic spindle cells, with adenocarcinoma components in the upper left corner. Original magnification: 100×.

Cholangiolocarcinoma (CLC) is a variant of iCCA that belongs to the small duct type. It is defined as an iCCA with more than 80% of the tumor area showing cholangiolocellular differentiation without hepatocellular differentiation. The prefix “cholangiolo” implies histopathological similarity to the cholangiole or canals of Hering (47). CLCs show small cuboidal cells forming cords or tubular structures with antler-like growth resembling the ductular reaction of non-tumorous liver (48). Often, the lumina of tumor cords are inconspicuous, the atypia or pleomorphism of tumor epithelial cells is minimal, and regularly spaced intervening stroma is also a characteristic feature (Figure 3A).

CLC is thought to arise at the bile ductule, containing hepatic stem or progenitor cells, and canals of Hering. It was previously classified as a subtype of combined hepatocellular-cholangiocarcinoma (49), however, molecular profiling studies favor the classification of CLC as part of iCCA (50). According to the current WHO classification, CLC without components of HCC or intermediate carcinoma is an iCCA and is not considered as combined hepatocellular-cholangiocarcinoma.

CLC is distinguished from conventional small duct type iCCAs based on its excellent outcome, which shows significantly higher overall and disease-free survival (49, 51). Even iCCAs with cholangiolocellular differentiation (>10% of the tumor area) were found to have a better prognosis than those without (17, 52). A transcriptomic profiling study reported that iCCA with cholangiolocellular differentiation correlated with inflammation class, while iCCA without cholangiolocellular differentiation correlated with proliferation class (the molecular classification is discussed in more detail later in the genomic-transcriptomic profiles section) (52, 53).

#### Intrahepatic Cholangiocarcinoma With Ductal Plate Malformation Pattern

Ductal plate malformation (DPM) refers to a developmental anomaly characterized by pathologically existing embryonic bile duct structures (“ductal plates”). The percentage of iCCAs that are diagnosed as iCCAs with DPM pattern is very small, approximately 2.9% of cases in a cohort of 175 resected iCCAs (54). Histopathologically, the tumor epithelial cell lining is usually benign-looking cuboidal cells without mucin production, and they form glandular structures that are elongated, tortuous, and coalesced, mimicking ductal plates (Figure 3B) (55). Genetic alterations in iCCA with DPM pattern include point mutations in *FGFR2*, *PTPRT*, *ARID1A*, and *CDKN2A*, and fusion of *FGFR2* (54, 56). Patient survival seems better than that of conventional small duct type iCCAs (54).

#### Adenosquamous Carcinoma/Squamous Carcinoma

Adenosquamous carcinoma of the liver has both squamous epithelial and glandular components (Figure 3C), and its incidence is rare (57). Squamous carcinoma, showing squamous differentiation in the entire tumor is extremely rare. This type of variant iCCA is reported to be correlated with chronic cholangitis caused by liver flukes or hepatolithiasis (58). The prognosis of adenosquamous carcinoma of the liver is usually poor, with a median survival of approximately 6 months (57).

#### Mucinous Carcinoma/Signet Ring Cell Carcinoma

Mucinous carcinoma is a variant that belongs to large duct type iCCA. It contains an overwhelming amount of extracellular mucin in the luminal space of tumor glands, usually over 50% of the total tumor volume by convention (59), often causing tumor epithelial cells to float in the mucin pool (Figure 3D). This type of tumor usually occurs due to the malignant transformation of the IPNB. Signet ring cell carcinoma occasionally presents as a mucinous carcinoma with varying distribution; however, pure signet ring cell carcinoma of the liver is extremely rare. The absence of ovarian-like stroma differentiates this variant of iCCA from mucinous cystic neoplasm (60).

#### Clear Cell Carcinoma

Clear cell carcinoma is characterized by bulky cytoplasmic clearing and eccentrically located nuclei in most tumor epithelial cells with glandular and trabecular growth patterns (Figure 3E) (58, 61). Primary clear cell carcinoma of the liver can be differentiated from HCC with clear cell change, metastatic clear cell carcinoma of the kidney, and metastasis from other gastrointestinal tract tumors by IHC staining for hepatocyte paraffin 1 (HepPar-1), CD10, and CK20, respectively (62).

#### Mucoepidermoid Carcinoma

Primary mucoepidermoid carcinoma of the liver shows features similar to those in other organs, including the salivary glands. It reveals a more intimate mixture of epidermoid or squamous and mucin-secreting elements, compared to adenosquamous carcinoma where mucin-secreting cells and foci of squamous differentiation exist separately (Figure 3F) (63). There have been only a few reports, and most of them have shown a poor prognosis (64).

#### Lymphoepithelioma-Like Carcinoma

Lymphoepithelioma-like carcinoma is characterized by dense lymphoid stroma around the tumor epithelial cells, often forming lymphoid follicles. Tumor epithelial cells show an undifferentiated or gland-forming pattern, rarely with well-differentiated or bland-looking glands (Figure 3G). Almost all cases are Epstein–Barr virus-encoded small RNA (EBER) positive and usually have favorable outcomes (65, 66).

#### Sarcomatous Intrahepatic Cholangiocarcinoma

Sarcomatous iCCA usually shows mixed features of conventional iCCA and undifferentiated components of cells with spindle or rhabdoid features (Figure 3H). When a conventional iCCA component is not present, a definite diagnosis is difficult, since the sarcomatoid component is often negative for epithelial markers by IHC staining (67). Sarcomatous iCCA usually has a worse prognosis than conventional iCCAs (68).

### Precursor Lesions

#### Biliary Intraepithelial Neoplasia

Biliary intraepithelial neoplasia (BilIN), a precursor lesion of CCA, occurs at the epithelium of intra- and extrahepatic bile ducts and in the peribiliary glands. Large duct type iCCA, but not small duct type, is often accompanied by BilIN (17, 21). BilIN is virtually invisible upon gross examination, although it may be associated with subtle changes such as mucosal thickening. Microscopically, BilIN consists of flat or micropapillary (less than 3 mm in height) epithelial lesions that are graded as low-grade or high-grade (carcinoma *in situ*) based on the highest degree of cytoarchitectural atypia (69, 70). This two-tiered classification replaces the former three-tiered classification, wherein the former BilIN-1 and BilIN-2 are now classified as low-grade, and the former BilIN-3 is now classified as high-grade.

**FIGURE 4:**
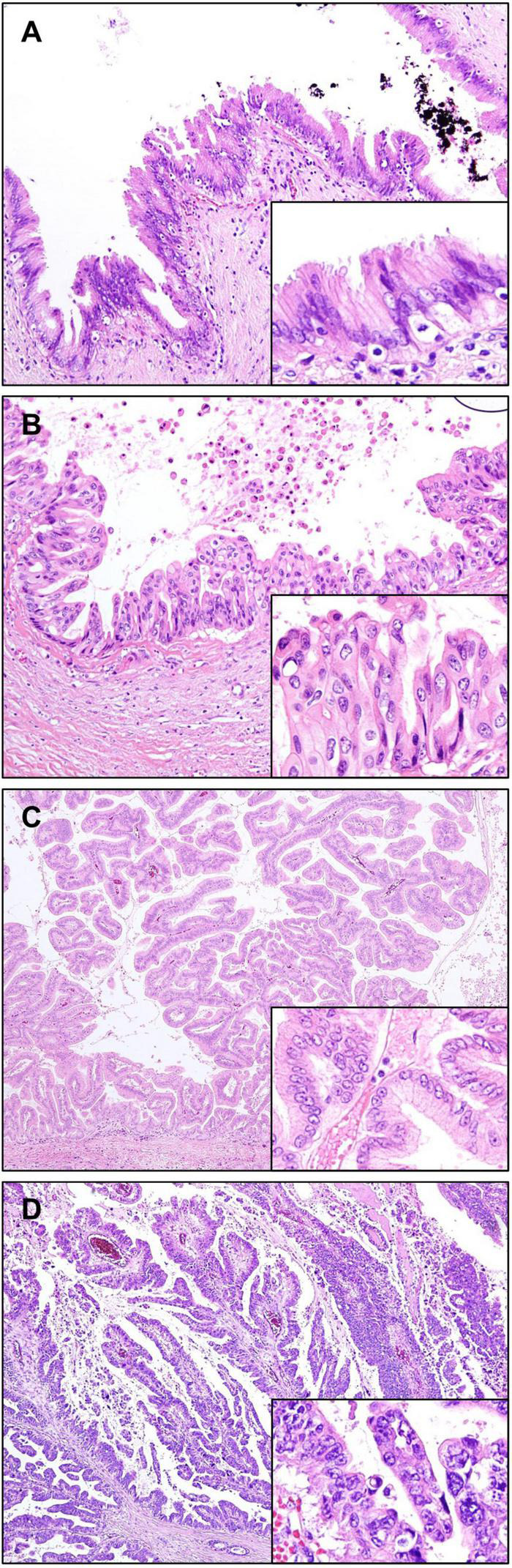
Precursor lesions of intrahepatic cholangiocarcinoma. **(A,B)** Biliary intraepithelial neoplasia (BilIN). **(A)** Low-grade BilIN composed of columnar cells with intact nuclear polarity and minimal atypia. **(B)** High-grade BilIN showing stratification of cells with marked nuclear atypia and loss of polarity. **(C,D)** Intraductal papillary neoplasm of the bile ducts (IPNB). **(C)** Low-grade IPNB showing a papillary growth of columnar biliary type epithelial cells with mild pleomorphism and preserved nuclear polarity. **(D)** High-grade IPNB showing irregular papillary projections, composed of highly pleomorphic and stratified cells with increased nuclear-cytoplasmic ratio. Original magnification: 100× for **(A,B)**, 40× for **(C,D)**, 200× for inset images.

Low-grade BilIN shows mild cytoarchitectural atypia, including flat pseudopapillary and/or micropapillary growth pattern, nuclear stratification, hyperchromatic nuclei, and increased nuclear-cytoplasmic ratio; however, nuclear polarity is preserved. High-grade BilIN is characterized by moderate to severe cytoarchitectural atypia, including more complex patterns, complete loss of polarity, marked nuclear atypia, and frequent mitosis. While IHC staining for p53 is usually negative in low-grade BilIN, it is often overexpressed in high-grade BilIN (71). The expression of p16 is relatively preserved in low-grade BilIN and decreased in high-grade BilIN (72). A notable mutation in BilIN lesions is alterations in *KRAS*, which is reported to be approximately 30% (73) (Figures 4A,B).

Differentiating BilIN from reactive epithelial atypia may be difficult, especially in biopsy samples. Reactive atypia shows overlapping attenuated basophilic cells with nuclei having fine and diffuse chromatin. The nucleoli are small or conspicuous. Mitotic activity may be prominent. Reactive epithelial atypia usually shows a gradual transition from uninvolved epithelium, in contrast to the abrupt change usually seen in BilIN. The IHC detection of S100P was shown to be useful, being mostly negative in reactive epithelial atypia. However, its expression increased sequentially from low-grade BilIN to high-grade BilIN and subsequently in iCCA (74).

#### Intraductal Papillary Neoplasm of the Bile Duct

Intraductal papillary neoplasm of the bile duct (IPNB) is defined as a grossly visible premalignant neoplasm showing intraductal papillary or villous growth of biliary-type epithelium (70). It is considered to be a counterpart of a similar tumor arising in the pancreas, the so-called intraductal papillary mucinous neoplasm (IPMN). IPNB is divided into low-grade and high-grade based on the highest degree of cytoarchitectural atypia. When invasive carcinoma develops in this lesion, it is diagnosed as IPNB with associated invasive carcinoma. High-grade IPNBs are often associated with stromal invasive carcinoma, usually consisting of tubular adenocarcinoma and occasionally mucinous carcinoma (75).

Grossly, IPNBs appear as polypoid masses with dilatation of the bile ducts. These are usually isolated papillary lesions, whereas some IPNBs appear as multiple contiguous papillary or polypoid lesions. Some IPNBs are characterized by mucus hypersecretion, forming mucin-containing fusiform dilatation or cysts, similar to those observed in IPMNs (76). Microscopically, IPNBs form papillary structures with fine fibrovascular cores (Figures 4C,D). Four histological subtypes are generally accepted based on cytological appearance and immunophenotype, namely, – pancreatobiliary, intestinal, gastric, and oncocytic. Immunohistochemically, MUC1 is mostly expressed in the pancreatobiliary type. Gastric type usually express MUC5AC and MUC6, and the intestinal type frequently express MUC2. CK20 is positive in the intestinal type, but not in the gastric and oncocytic types (70, 77). The presence of two or more histopathological types is common in IPNB, therefore these tumors are diagnosed based on the most prevalent histopathological type. The pancreatobiliary type is most common, with higher prevalence in western countries than in Asia. In contrast, intestinal type is more common in Asian populations than in western populations, while oncocytic and gastric types are least frequent (78). Although the clinical implications of histopathologic subtypes are still controversial, the pancreatobiliary type is reported to be linked with a higher frequency of associated invasive carcinoma, frequent lymph node metastasis, and recurrence (77).

A recent consensus has proposed a different classification for IPNBs of type 1 and type 2 (79, 80). Type 1 IPNB shows more homogeneous appearance than type 2 IPNB, and is composed of regular villous, papillary, or tubular structures usually with low-grade dysplasia, but may present with high-grade dysplasia with foci of low-grade dysplasia. Mucin overproduction is frequently observed, whereas stromal invasion is uncommon. This is most commonly found in intrahepatic bile ducts. Histological similarity with IPMN of the pancreas is also characteristic. Type 2 IPNB exhibits heterogenous appearance composed of irregular and complicated villous, papillary or tubular structures. This is usually composed of high-grade dysplasia, and foci of low-grade dysplasia are absent or minimal. Invasive carcinoma is more frequently associated with type 2 IPNB than type 1 IPNB. Mucin overproduction is not common. Type 2 IPNB arises throughout the biliary tree, including intrahepatic and extrahepatic bile ducts.

The mutational profile studies on IPNB have reported that diverse cancer driver mutations are frequently observed, including *KRAS*, *TP53*, *STK11*, *CTNNB1*, *APC*, *SMAD4*, and *GNAS*. Type 1 IPNBs show higher mutation rates of *KRAS*, *GNAS*, and *RNF43*, whereas type 2 IPNBs have higher *TP53* and *SMAD4* mutation rates (81, 82).

Although IPNBs present papillary morphology, sometimes tubular growth pattern of epithelial components with less mucin production is predominantly observed, similar to intraductal tubulopapillary neoplasm (ITPN) of the pancreas (83). Such cases have been described as intraductal tubular neoplasms or ITPNs. Recently, ITPN has been reported to show more frequent intrahepatic occurrence in contrast to IPNB, which favors both intra- and extrahepatic locations. Furthermore, IPNB and ITPN differ in their genomic and epigenomic profiles. Recently, IPNB has been reported to share mutational profiles with extrahepatic CCA, including mutations in *TP53*, *SMAD4*, and *KRAS* and deletions on chromosomes 9q, 17p, and 18q. However, ITPN shows low overall mutational burden, and distinct DNA methylation pattern that clustered together with iCCA rather than extrahepatic CCA, suggesting that IPNB and ITPN are distinct entities (84).

## Tumor Microenvironment of Intrahepatic Cholangiocarcinoma

**FIGURE 5:**
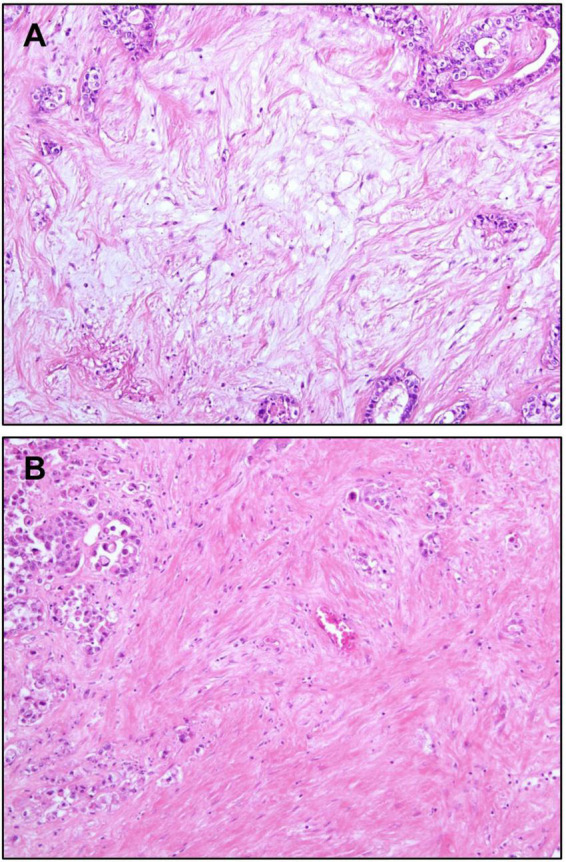
Stromal features of intrahepatic cholangiocarcinoma. **(A)** Immature stroma showing pale basophilic myxoid appearance. Activated fibroblasts are the main components of the stroma. Collagen fibers are incomplete and thin. **(B)** Mature stroma showing thick collagen bundles, making its eosinophilic color. Original magnification: 100×.

One of the characteristics of iCCA is the abundance of fibrous stroma (85). The amount of fibrous stroma has been reported to be associated with poor prognosis (86). Furthermore, so-called “scirrhous type” iCCA, which is defined as iCCA having scirrhous area (where the amount of fibrous stromal component is at least equal to the area of epithelial component) more than 70% of the largest cut surface, has been reported to show worse prognosis than conventional iCCAs (87). More recently, the characteristics of immature and mature fibrous tumor stroma have been reported to be related to tumor behavior (88, 89). Immature stroma is composed of myxoid stroma with randomly oriented short keloid-like collagen bundles. In contrast, mature stroma shows multilayered mature collagen fibers (Figure 5). Accordingly, iCCAs with immature stroma have been reported to show poorer prognosis compared to iCCAs with mature stroma (88, 89).

Activated CAFs, one of the major components of the tumor microenvironment, have been demonstrated to facilitate tumor growth and progression, and promote immunosuppression in tumors (90). CAFs are thought to be recruited from hepatic stellate cells, portal fibroblasts, or circulating mesenchymal cells, but the exact source is currently unknown (91). Transforming growth factor β (TGF-β) and platelet-derived growth factor D (PDGF-D) secreted by tumor epithelial cells recruit CAFs. Recruited CAFs not only promote desmoplastic reaction by collagen and matrix metalloproteinases, but also cause tumor epithelial cells to proliferate, invade, and resist antitumor mechanisms by secreting growth factors such as PDGF-B and epidermal growth factors (92, 93). Patients with iCCA with a high proportion of activated CAFs were reported to have a shorter survival rate than patients with low CAF proportion (94).

Immune cells, including tumor-associated macrophages (TAMs) and tumor-infiltrating lymphocytes, are also main components of the tumor microenvironment. The hepatic macrophage population consists of activated macrophages derived from Kupffer cells or bone marrow-derived macrophages (95), and activated macrophages can be classified as M1- (classical) and M2 (alternative)-polarized types (96). The M2 phenotype forms the majority of TAM population in iCCA, having anti-inflammatory and pro-tumor functions mediated by the secretion of anti-inflammatory cytokines, including IL-4, IL-10, IL-13, and TGF-β (97). These cells also promote intratumoral angiogenesis, which is vital for tumor survival and metastasis (85, 98). A high proportion of M2 TAMs in iCCA is correlated with increased invasiveness of tumor cells and poor disease-free survival (97, 99). In contrast, M1 TAMs have been reported to exert pro-inflammatory functions, including secretion of pro-inflammatory cytokines such as TNF-α, interleukin (IL)-6, and IL-1β (99).

Tumor-infiltrating lymphocytes include B cells (CD20^+^), helper T cells (CD4^+^), cytotoxic T cells (CD8^+^), and regulatory T cells (Tregs, FOXP3^+^). The major proportion of tumor-infiltrating lymphocytes comprises T cells rather than B cells (100). The distribution and proportion of CD4^+^ and CD8^+^ T cells varies among iCCAs, and increased population of these cells is correlated with better prognosis (8, 85). In addition, iCCAs with B cell infiltration have been reported to be associated with better survival than iCCAs without B cells (101). Treg cells are a subset of CD4^+^ T cells that suppress innate and adaptive immune responses mainly by secreting IL-10 and TGF-β, which are known to promote tumor progression by inhibiting antitumor immune response. Regarding iCCA, while there are a few studies on the clinical aspects of the presence of Treg cells, there is no sufficient evidence to draw a conclusion. Therefore, additional investigation is required (101–103).

With the advent of immune checkpoint blockade therapeutics, the expression status of cell surface proteins with immune escape mechanisms is currently under active investigation. Cytotoxic T-lymphocyte antigen-4 (CTLA-4), expressed on the surface of Treg cells, suppresses cytotoxic T cell activity by binding to CD80 of antigen-presenting cells (85). High CTLA-4 expression has been reported to be related to worse relapse-free survival of patients with CCA, raising the possibility of effective immunotherapy targeting CTLA-4 (104). Programmed death 1 (PD-1), expressed on T cells, and its ligand programmed death ligand 1 (PD-L1), on tumor epithelial cells, are other major immune checkpoints of interest. Binding of PD-L1 to PD-1 diminishes the immunological function of cytotoxic T cells. Approximately 9–30% of iCCA has been reported to be PD-L1 positive as observed by IHC staining (105–107). Recently, our group reported that *KRAS* alteration and chromosomal instability were associated with resistance to PD-1/PD-L1 blockade immunotherapy, whereas high intratumoral tumor-infiltrating lymphocyte density was associated with a favorable immunotherapy response in patients with CCA (35). Many clinical trials for iCCA using immune checkpoint inhibitors are ongoing based on the expression status of markers, including PD-1, PD-L1, and CTLA-4, with expectations of promising results in the near future (108).

## Multi-Omics Features of Intrahepatic Cholangiocarcinoma

### Germline and Somatic Mutational Profile of Intrahepatic Cholangiocarcinoma

#### Germline Predisposition

There are a few germline predispositions for cancers, including proto-oncogenes and tumor suppressor genes, either by inheritance from parents or *de novo* mutation at the zygote level. Approximately 8–12% of iCCAs have been reported to have known pathogenic or possibly deleterious germline mutations, and the most commonly found germline-mutated genes are *BRCA1* and *BRCA2*, which are associated with DNA repair mechanism and hereditary cancer syndromes (109–112) (Table 1). Other germline variants linked with iCCA include *APC*, an antagonist of the Wnt signaling pathway, *BAP1*, which mediates deubiquitination, and mismatch repair mechanism-related genes, namely *MLH1* and *MSH2* (109, 111, 113). However, evidence regarding the association between known hereditary cancer syndromes and iCCA is currently not fully established and requires further investigation.

**TABLE 1:** Summary of germline mutations reported in intrahepatic cholangiocarcinoma.

Gene	Frequency of occurrence (%)	References
*BRCA1*	1–3	(109–111)
*BRCA2*	1–3	(109–111)
*MLH1*	2	(109)
*MSH2*	2	(109)
*MUTYH*	2	(111)
*BAP1*	1	(111)
*PMS2*	1	(111)
*APC*	1	(111)

#### Somatic Short Mutations, Structural Variations, and Copy Number Aberrations

Among somatic mutations identified in iCCA, the most well-known and frequent variants are at exons 5–8 of *TP53* and hotspots at codons 12/13 of *KRAS*, which are involved in cell cycle arrest/DNA repair and mitogen-activated protein kinase (MAPK) signaling pathway, respectively (114–116). Other MAPK pathway genes such as *NRAS* and *BRAF* are also frequently mutated in iCCA (117). Owing to the advancement and wide use of massive parallel sequencing techniques, many other driver gene mutations have been discovered in the last decade. Single-nucleotide variants of chromatin remodeling-related genes such as *ARID1A*, *BAP1*, and *PBRM1* have been reported with frequencies ranging from 6 to over 30% (115, 118–120). Moreover, mutations in *IDH1/2*, which acts as an epigenetic regulator, are most frequently observed, with an average incidence of approximately 15% (115, 121–124). Other somatic short mutations include Akt signaling pathway-associated genes such as *PTEN*, *PIK3CA*, and *PIK3C2A*, and *SMAD4*, a TGF-β signaling pathway gene (115, 118, 125, 126).

The most frequently found structural variation in iCCA is *FGFR* gene fusion, notably *FGFR2*, which has been reported in 6–14% of iCCAs (127, 128). The most common fusion partner is *BICC1*; however, several other genes were also found, including *AHCYL1* and *PPHLN1* (128–130). Driver gene amplification was found in *ERBB2* (2–12%), *MDM2* (0–13%), *EGFR* (1–16%), and *CCND1* (10–13%). Deletion of 9p21.3, or the locus including genes *CDKN2A* and *CDKN2B* is found in 10–20% of iCCA (13, 126, 127, 131).

Microsatellite instability-high cases are usually determined by three methods namely, observing the size change in more than three out of five marker loci by polymerase chain reaction, IHC for mismatch repair proteins including, MLH1, MSH2, MSH6, and PMS2, or estimation of tumor mutation burden by NGS. Such cases are known to be rare (around 1%) in iCCA (126, 132–134). The mutational characteristics of iCCA are summarized in Table 2.

**TABLE 2:** Major somatic variants and reported incidence in intrahepatic cholangiocarcinoma.

	Groups	Gene or locus	Frequency of occurrence (range, %)	References
Small nucleotide variants	DNA repair	*TP53*	2.5–39.3	(13, 35, 115, 120, 123, 125, 126, 131)
	Chromatin remodeling	*ARID1A*	7–36	(13, 35, 115, 120, 123, 126, 131)
		*BAP1*	6–16	(13, 35, 116, 121, 123, 126, 131)
		*PBRM1*	9–14.3	(13, 35, 116, 119, 121, 123, 126, 131)
	MAPK signaling pathway	*KRAS*	2–30.3	(13, 35, 115, 121, 123, 125, 126, 131)
		*NRAS*	3–9.3	(13, 115, 117, 119, 125, 126, 131)
		*BRAF*	3–5	(13, 35, 117, 125, 126, 131)
	Epigenetic regulator	*IDH1*	5–36	(13, 35, 115, 126, 131)
		*IDH2*	3.7–36	(13, 115, 117, 119, 121, 126, 131)
	TGF-β signaling pathway	*SMAD4*	0–9	(13, 35, 115, 126, 131)
	Akt signaling Pathway	*PTEN*	0.6–11	(13, 115, 117, 125, 126, 131)
		*PIK3CA*	3–7	(13, 35, 115, 117, 119, 120, 125, 126, 131)
		*PIK3C2A*	0–7.1	(117)
Structural variation	Translocation	*FGFR2*	6–14	(13, 127, 128, 131)
	Amplification	*CCND1*	10–13	(13, 35, 131)
		*EGFR*	1–16	(126, 131)
		*ERBB2*	2–12	(35, 126, 131)
		*MDM2*	0–13	(131)
	Deletion	9p21.3 (CDKN2A/B)	10–20	(126, 131)
Microsatellite instability			∼1	(126, 131, 133)

Genetic alterations are also correlated with pathological features. Hotspot mutations in *KRAS* have been reported in periductal infiltrating type, but not in mass-forming type (135). Histopathologically, small duct type has been reported to have more frequent *BAP1* and *IDH1/2* hotspot mutations and *FGFR2* fusion, and lower incidence of *KRAS* mutation than large duct type (12, 26, 51, 122, 136, 137). On the contrary, large duct type is known to have frequent mutations in *TP53*, *KRAS* and some TGF-β pathway genes, including *SMAD4*, *TGFBR2*, *FBXW7*, and *MYC* (35).

From an etiological point of view, liver fluke *O. viverrini* infection-related iCCA had a higher *TP53* mutation rate, while *BAP1* and *IDH1/2* mutations were more frequently found in non-fluke-related cases (131, 138). *TP53* mutation was also found to be significantly correlated with hepatitis B virus (HBV) infection (127, 139). Regarding patient outcome, worse overall survival of patients with mutated *TP53*, *KRAS*, and *TERT* or deleted *CDKN2A* has been reported (126).

### Genomic-Transcriptomic Profiles: Molecular Classification of Intrahepatic Cholangiocarcinoma

Several multi-omics approaches have been reported in the past decade, and several molecular classifications of iCCA have been presented (Table 3).

**TABLE 3:** Notable classification of intrahepatic cholangiocarcinoma from multi-omics studies.

Base of classification	Number of cases	Molecular classification and characteristics	References
Inflammation versus proliferation signature	149	• Inflammation class - Enriched in immune response-related pathways - Overexpression of *IL-4* and *IL-10* (Th2 marker) - Favorable prognosis • Proliferation class - Enriched in oncogenic pathways including RTK and angiogenic pathways, increased expression of *EGF*, *RAS*, *AKT*, *MET*, and growth factors - Worse outcome compared to inflammation class	Sia et al. (53)
Prognosis	104*	• Cluster 1 (group with good prognosis) - No *KRAS* mutation - Absence or weak expression of *HER2* and *MET* • Cluster 2 (group with poor prognosis) - Enriched *VEGF*/*ERBB*, *CTNNB1*/*MYC*, and TNF pathway and *KRAS* mutation	Andersen et al. (140)
Tumor microenvironment	78	• Immune desert subtype - Minimal expression of all TME signatures • Immunogenic subtype - High innate and adaptive immune cell presence - Strong activation of fibroblasts and inflammatory and immune checkpoint pathways - Best outcome • Myeloid-rich subtype - Strong monocyte-derived myeloid cell signatures - Low lymphoid signatures • Mesenchymal subtype - Strong active fibroblast signatures - Worst outcome	Job et al. (142)
TCGA project	32	• *IDH*-mutant cluster - *IDH1/2* mutation - Enriched mitochondrial gene expression - Loss of function of *ARID1A* and *PBRM1* • *CCND1* amplification cluster - Highly methylated - *BAP1*/*FGFR* cluster • *BAP1* mutation or *FGFR2* fusion • Survival difference is not significant between clusters	Farshidfar et al. (13)
Etiologic factor-associated	69	• Cluster 1 - Liver fluke-related - *ARID1A*, *BRCA1/2*, and *TP53* mutations - *ERBB2* amplification - CpG island hypermethylation • Cluster 2 - Partly liver-fluke-related - *TP53* mutation - High expression of *CTNNB1*, *WNT5B* and *AKT1* • Cluster 3 - High CNA burden - Enriched immune-related pathways • Cluster 4 - Associated with viral hepatitis - *BAP1* or *IDH1/2* mutation - High expression of *FGFR* family proteins - CpG shore hypermethylation - Favorable prognosis	Jusakul et al. (131)

*Whether only intrahepatic cholangiocarcinoma was included is not certain.

*CNA, copy number aberration; HCC, hepatocellular carcinoma; IL, interleukin; RTK, receptor tyrosine kinase; TCGA-CHOL, The Cancer Genome Atlas-Cholangiocarcinoma Consortium; TME, tumor microenvironment.*

#### Inflammation/Proliferation Class

Integrated gene expression and mutational analyses performed by Sia et al. revealed two classes (inflammation and proliferation) of iCCA (53). The inflammation class accounted for approximately 40% of iCCA, and it was characterized by activation of immune response-related pathways, including dendritic cell signature and cytokines such as IL-4 and IL-10. The proliferation class showed activation of several oncogenic pathways including receptor tyrosine kinase pathway genes, such as *EGF*, *RAS*, *AKT*, *MET*, and other growth factor genes. Patient outcomes were worse in the proliferation class than in the inflammation class.

#### Prognosis-Based Classes

Transcriptomic profiling of iCCA and perihilar CCA by Andersen et al. revealed two prognostic groups (C1 and C2) with 5-year survival rate. The group with poor prognosis (C2) indicated increased activation of *VEGF*/*ERBB*, *CTNNB1*/*MYC*, and *TNF* signaling network and *KRAS* mutation, whereas these characteristics were not seen in the group with good prognosis (C1) (140). Recently, the gene expression pattern of iCCAs with cholangiolocellular differentiation trait, having favorable prognosis, was reported to be similar to that of C1, and has a signature including upregulated expression of inflammation-related genes and downregulated expression of proliferation-related genes based on Gene Ontology terms (52, 141).

#### Tumor Microenvironment-Based Classes

A recent study on the classification of iCCA according to its tumor microenvironment presented four subtypes based on gene signature analysis: “immunogenic,” which shows high innate and adaptive immune cell infiltration, “myeloid-rich,” which has strong macrophage and myeloid signatures, “mesenchymal,” with strong activated fibroblast signature, and “immune-desert,” which is characterized by lowest expression of all signatures (142). The immunogenic subtype had the best outcome, whereas the mesenchymal subtype had the worst outcome, in agreement with the prognostic features of the tumor microenvironment of iCCA discussed previously.

#### Other Classifications

Multi-omics data from TCGA project revealed that *IDH1/2-* and *PBRM1*-mutant subgroups showed upregulation of mitochondrial genes and downregulation of chromatin-modifying genes such as *ARID1A* and *ARID1B* due to hypermethylation of the promoter CpG region, while cases with *FGFR2* fusion showed downregulation of mitochondrial genes (13). Furthermore, another study has proposed a classification based on the correlation of multi-omics features with etiologic background, showing that liver fluke-associated clusters 1 and 2, which harbor *TP53* mutation and *ERBB2* amplification in common, can be differentiated based on hypermethylated CpG island (for cluster 1). Furthermore, liver fluke-negative clusters 3 and 4 can be subdivided based on immune-related pathway enrichment (for cluster 3), and *IDH1/2* mutations, *FGFR2* fusion, and hypermethylated CpG promoter shores associated with viral hepatitis (for cluster 4) (131).

### Moleculo-Pathological Correlation

**FIGURE 6:**
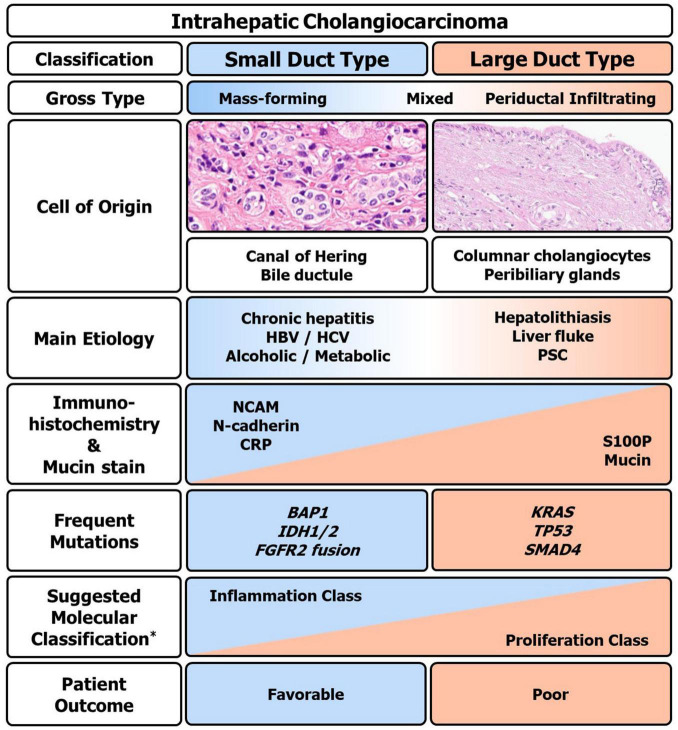
Clinico-pathologic and molecular summary of intrahepatic cholangiocarcinoma (iCCA). Macro and microscopic, immunohistochemical, mutational, and clinical overview of iCCA. HBV, hepatitis B virus; HCV, hepatitis C virus; PSC, primary sclerosing cholangitis; S100P, S100 calcium-binding protein P; NCAM, neural cell adhesion molecule; CRP, c-reactive protein. *Based on the classification by Sia et al. (53).

The two major pathological types, small duct type and large duct type iCCA differ in their molecular characteristics. Small duct type iCCAs frequently have *BAP1*, *IDH1/2*, and *FGFR* mutations, while large duct type iCCAs more commonly show *KRAS*, *TP53*, and *SMAD4* mutations. Interestingly, iCCA with cholangiolocellular differentiation trait, which belongs to the small duct type, has been reported to be correlated with inflammation class and group with good prognosis (C1) (52, 53, 140). The pathological, clinical, and molecular characteristics of iCCA based on currently available evidence are summarized in Figure 6.

### Perspectives on Targeted Therapies

Large-scale genomic analyses have identified target molecules for chemotherapy of patients with iCCA. Thus far, use of fibroblast growth factor receptor (FGFR) inhibitors and isocitrate dehydrogenase (IDH) 1 and 2 inhibitors are promising strategies against iCCA (130, 143).

Fibroblast growth factor receptor family proteins are localized on the cell membrane and transfer extracellular growth signals through intracellular tyrosine kinase domains. Pemigatinib, an oral inhibitor of FGFR1-3, has been approved by the United States Food and Drug Administration (FDA) for the treatment of patients with refractory advanced CCA with *FGFR2* fusion (130).

Isocitrate dehydrogenase 1 and 2 proteins are essential components of the tricarboxylic acid cycle, that normally generate NADPH *via* conversion of isocitrate into α-ketoglutarate. Mutant IDH1/2 proteins accelerate this process, resulting in excess production of the byproduct, 2-hydroxyglutarate, which acts as an oncometabolite by interfering with histone and DNA methylation regulation (143). A recent phase 3 clinical trial of the IDH1 inhibitor ivosidenib in patients with advanced iCCA has shown promising results in increasing overall survival, and the FDA has approved its use in previously treated *IDH1*-mutated iCCA patients (143, 144).

Even though there is hope for these approved target agents, application is limited to those who harbor specific mutations, and antitumor efficacy is limited by intratumoral heterogeneity and drug resistance. Other targeted protein inhibitor molecules are in active clinical trials, including other inhibitors of FGFR family; multi-kinase inhibitors that act on epidermal growth factor receptor, vascular endothelial growth factor receptor and platelet-derived growth factor receptor; specific tyrosine kinase inhibitors targeting HER2, BRAF inhibitors, and immune checkpoint inhibitors (145, 146). To detect a variety of potential actionable mutations, the European Society for Medical Oncology has recommended routine use of NGS in patients with advanced cholangiocarcinoma (147).

## Conclusion

Intrahepatic cholangiocarcinoma is a very heterogenous malignancy with respect to histomorphology and molecular perspectives. The tumor microenvironment of iCCA also varies significantly depending on the type of immune cell infiltration and tumor stromal characteristics. Histopathological classification of small duct and large duct types shows differences in etiology, molecular features, and clinical outcomes. Analyses of NGS and multi-omics studies have suggested molecular classifications of iCCA and identified *FGFR2* fusion and *IDH1/2* mutations as indications for targeted drugs. Further studies are needed for better pathological-molecular correlation and marker development for targeted therapy as well as immunotherapy to improve the treatment efficacy of patients with iCCA.

## Author Contributions

TC and YP contributed to the conception and design of this manuscript. TC performed the literature search, data analysis, and wrote the first draft of the manuscript. YP critically revised the manuscript. Both authors approved the submitted version.

## Conflict of Interest

The authors declare that the research was conducted in the absence of any commercial or financial relationships that could be construed as a potential conflict of interest.

## Publisher’s Note

All claims expressed in this article are solely those of the authors and do not necessarily represent those of their affiliated organizations, or those of the publisher, the editors and the reviewers. Any product that may be evaluated in this article, or claim that may be made by its manufacturer, is not guaranteed or endorsed by the publisher.

## Abbreviations

CCA: cholangiocarcinoma
iCCA: intrahepatic cholangiocarcinoma
CAFs: cancer-associated fibroblasts
TCGA: The Cancer Genome Atlas
WHO: World Health Organization
MUC: mucin
CRP: c-reactive protein
EBER: Epstein–Barr virus-encoded small RNA
CKs: cytokeratins
FGB: fibrinopeptide B
CLC: cholangiolocarcinoma
HCC: hepatocellular carcinoma
DPM: ductal plate malformation
IPNB: intraductal papillary neoplasm of the bile duct
BilIN: biliary intraepithelial neoplasia
IPMN: intraductal papillary mucinous neoplasm
TAMs: tumor-associated macrophages.

## References

[1] Nagorney DM Pawlik TM Chun YS Ebata T Vauthey J-N. Perihilar bile ducts. 8th ed. In: Edge SB American Joint Committe on Cancer editors. *AJCC Cancer Staging Manual.* New York, NY: Springer (2017).

[2] Massarweh NN El-Serag HB. Epidemiology of Hepatocellular carcinoma and intrahepatic cholangiocarcinoma. *Cancer Control.* (2017) 24:1073274817729245. 10.1177/1073274817729245 28975830 PMC5937247

[3] Nakanuma Y Klimstra DS Komuta M Zen Y. Intrahepatic cholangiocarcinoma. 5th ed. In: WHO Classification of Tumours Editorial Board editor. *Digestive System Tumours.* Lyon: International Agency for Research on Cancer (2019).

[4] Florio AA Ferlay J Znaor A Ruggieri D Alvarez CS Laversanne M Global incidence of and trends in intrahepatic and extrahepatic cholangiocarcinoma from 1993 to 2012. *Cancer.* (2020) 126:2666–78. 10.1002/cncr.32803 32129902 PMC7323858

[5] Khan SA Tavolari S Brandi G. Cholangiocarcinoma: epidemiology and risk factors. *Liver Int.* (2019) 39:19–31. 10.1111/liv.14095 30851228

[6] Jeong Y-I Shin H-E Lee S-E Cheun H-I Ju J-W Kim J-Y Prevalence of *Clonorchis sinensis* Infection among residents along 5 major rivers in the republic of Korea. *Korean J Parasitol.* (2016) 54:215–9. 10.3347/kjp.2016.54.2.215 27180582 PMC4870967

[7] Kitphati R Watanawong O Wongsaroj T Nithikathkul C. National program of opisthorchiasis in Thailand; situation and policy strategy. *Int J Geoinf.* (2021) 17:61–8. 10.52939/ijg.v17i2.1759

[8] Banales JM Marin JJG Lamarca A Rodrigues PM Khan SA Roberts LR Cholangiocarcinoma 2020: the next horizon in mechanisms and management. *Nat Rev Gastroenterol Hepatol.* (2020) 17:557–88. 10.1038/s41575-020-0310-z 32606456 PMC7447603

[9] Buettner S van Vugt JL Jn IJ Groot Koerkamp B. Intrahepatic cholangiocarcinoma: current perspectives. *OncoTargets Ther.* (2017) 10:1131–42. 10.2147/ott.S93629 28260927 PMC5328612

[10] Yu T-H Chen X Zhang X-H Zhang E-C Sun C-X. Clinicopathological characteristics and prognostic factors for intrahepatic cholangiocarcinoma: a population-based study. *Sci Rep.* (2021) 11:3990. 10.1038/s41598-021-83149-5 33597569 PMC7889915

[11] Lee Y-T Wang JJ Luu M Noureddin M Nissen NN Patel TC Comparison of clinical features and outcomes between intrahepatic cholangiocarcinoma and hepatocellular carcinoma in the United States. *Hepatology.* (2021) 74:2622–32. 10.1002/hep.32007 34114675

[12] Hayashi A Misumi K Shibahara J Arita J Sakamoto Y Hasegawa K Distinct clinicopathologic and genetic features of 2 histologic subtypes of intrahepatic cholangiocarcinoma. *Am J Surg Pathol.* (2016) 40:1021–30. 10.1097/pas.0000000000000670 27259014

[13] Farshidfar F Zheng S Gingras MC Newton Y Shih J Robertson AG Integrative genomic analysis of cholangiocarcinoma identifies distinct IDH-mutant molecular profiles. *Cell Rep.* (2017) 18:2780–94. 10.1016/j.celrep.2017.02.033 28297679 PMC5493145

[14] Cerami E Gao J Dogrusoz U Gross BE Sumer SO Aksoy BA The cBio cancer genomics portal: an open platform for exploring multidimensional cancer genomics data. *J Cancer Discov.* (2012) 2:401–4. 10.1158/2159-8290.CD-12-0095 22588877 PMC3956037

[15] Yamasaki S. Intrahepatic cholangiocarcinoma: macroscopic type and stage classification. *J Hepatobiliary Pancreat Surg.* (2003) 10:288–91. 10.1007/s00534-002-0732-8 14598147

[16] Nakanuma Y Miyata T Uchida T. Latest advances in the pathological understanding of cholangiocarcinomas. *Expert Rev Gastroenterol Hepatol.* (2016) 10:113–27. 10.1586/17474124.2016.1104246 26492529

[17] Chung T Rhee H Nahm JH Jeon Y Yoo JE Kim Y-J Clinicopathological characteristics of intrahepatic cholangiocarcinoma according to gross morphologic type: cholangiolocellular differentiation traits and inflammation- and proliferation-phenotypes. *HPB.* (2020) 22:864–73. 10.1016/j.hpb.2019.10.009 31735647

[18] Kim Y Lee K Jeong S Wen X Cho NY Kang GH. DLEC1 methylation is associated with a better clinical outcome in patients with intrahepatic cholangiocarcinoma of the small duct subtype. *Virchows Arch.* (2019) 475:49–58. 10.1007/s00428-018-02511-7 30610381

[19] Sigel CS Drill E Zhou Y Basturk O Askan G Pak LM Intrahepatic cholangiocarcinomas have histologically and immunophenotypically distinct small and large duct patterns. *Am J Surg Pathol.* (2018) 42:1334–45. 10.1097/pas.0000000000001118 30001234 PMC6657522

[20] Aishima S Oda Y. Pathogenesis and classification of intrahepatic cholangiocarcinoma: different characters of perihilar large duct type versus peripheral small duct type. *J Hepatobiliary Pancreat Sci.* (2015) 22:94–100. 10.1002/jhbp.154 25181580

[21] Akita M Sofue K Fujikura K Otani K Itoh T Ajiki T Histological and molecular characterization of intrahepatic bile duct cancers suggests an expanded definition of perihilar cholangiocarcinoma. *HPB (Oxford).* (2019) 21:226–34. 10.1016/j.hpb.2018.07.021 30170977

[22] Komuta M Govaere O Vandecaveye V Akiba J Van Steenbergen W Verslype C Histological diversity in cholangiocellular carcinoma reflects the different cholangiocyte phenotypes. *Hepatology.* (2012) 55:1876–88. 10.1002/hep.25595 22271564

[23] Theise ND Saxena R Portmann BC Thung SN Yee H Chiriboga L The canals of Hering and hepatic stem cells in humans. *Hepatology.* (1999) 30:1425–33. 10.1002/hep.510300614 10573521

[24] Guest RV Boulter L Kendall TJ Minnis-Lyons SE Walker R Wigmore SJ Cell lineage tracing reveals a biliary origin of intrahepatic cholangiocarcinoma. *J Cancer Res.* (2014) 74:1005–10. 10.1158/0008-5472.CAN-13-1911 24310400 PMC3929349

[25] Moeini A Haber PK Sia D. Cell of origin in biliary tract cancers and clinical implications. *JHEP Rep.* (2021) 3:100226. 10.1016/j.jhepr.2021.100226 33665585 PMC7902553

[26] Tsai JH Huang WC Kuo KT Yuan RH Chen YL Jeng YM. S100P immunostaining identifies a subset of peripheral-type intrahepatic cholangiocarcinomas with morphological and molecular features similar to those of perihilar and extrahepatic cholangiocarcinomas. *Histopathology.* (2012) 61:1106–16. 10.1111/j.1365-2559.2012.04316.x 22882148

[27] Aishima S Kuroda Y Nishihara Y Taguchi K Taketomi A Maehara Y Gastric mucin phenotype defines tumour progression and prognosis of intrahepatic cholangiocarcinoma: gastric foveolar type is associated with aggressive tumour behaviour. *Histopathology.* (2006) 49:35–44. 10.1111/j.1365-2559.2006.02414.x 16842244

[28] Yu TH Yuan RH Chen YL Yang WC Hsu HC Jeng YM. Viral hepatitis is associated with intrahepatic cholangiocarcinoma with cholangiolar differentiation and N-cadherin expression. *Mod Pathol.* (2011) 24:810–9. 10.1038/modpathol.2011.41 21423153

[29] Kozaka K Sasaki M Fujii T Harada K Zen Y Sato Y A subgroup of intrahepatic cholangiocarcinoma with an infiltrating replacement growth pattern and a resemblance to reactive proliferating bile ductules: ‘bile ductular carcinoma’. *Histopathology.* (2007) 51:390–400. 10.1111/j.1365-2559.2007.02735.x 17553067

[30] Akita M Sawada R Komatsu M Suleman N Itoh T Ajiki T An immunostaining panel of C-reactive protein, N-cadherin, and S100 calcium binding protein P is useful for intrahepatic cholangiocarcinoma subtyping. *Hum Pathol.* (2021) 109:45–52. 10.1016/j.humpath.2020.12.005 33321161

[31] Vijgen S Terris B Rubbia-Brandt L. Pathology of intrahepatic cholangiocarcinoma. *Hepatobiliary Surg Nutr.* (2017) 6:22–34. 10.21037/hbsn.2016.11.04 28261592 PMC5332210

[32] Park JH Kim JH. Pathologic differential diagnosis of metastatic carcinoma in the liver. *Clin Mol Hepatol.* (2019) 25:12–20. 10.3350/cmh.2018.0067 30300991 PMC6435968

[33] Misumi K Hayashi A Shibahara J Arita J Sakamoto Y Hasegawa K Intrahepatic cholangiocarcinoma frequently shows loss of BAP1 and PBRM1 expression, and demonstrates specific clinicopathological and genetic characteristics with BAP1 loss. *Histopathology.* (2017) 70:766–74. 10.1111/his.13127 27864835

[34] Jeon Y Kwon SM Rhee H Yoo JE Chung T Woo HG Molecular and radiopathologic spectrum between HCC and intrahepatic cholangiocarcinoma. *Hepatology* (2022). 10.1002/hep.32397. [Epub ahead of print]. 35124821

[35] Yoon JG Kim MH Jang M Kim H Hwang HK Kang CM Molecular characterization of biliary tract cancer predicts chemotherapy and PD-1/PD-L1 blockade responses. *Hepatology.* (2021) 74:1914–31. 10.1002/hep.31862 33884649

[36] Choi W-T Ramachandran R Kakar S. Immunohistochemical approach for the diagnosis of a liver mass on small biopsy specimens. *Hum Pathol.* (2017) 63:1–13. 10.1016/j.humpath.2016.12.025 28087475

[37] Werling RW Yaziji H Bacchi CE Gown AM. CDX2, a Highly sensitive and specific marker of adenocarcinomas of intestinal origin: an immunohistochemical survey of 476 primary and metastatic carcinomas. *Am J Surg Pathol.* (2003) 27:303–10. 10.1097/00000478-200303000-00003 12604886

[38] Stenhouse G Fyfe N King G Chapman A Kerr KM. Thyroid transcription factor 1 in pulmonary adenocarcinoma. *J Clin Pathol.* (2004) 57:383–7. 10.1136/jcp.2003.007138 15047742 PMC1770279

[39] Surrey LF Frank R Zhang PJ Furth EE. TTF-1 and Napsin-A are expressed in a subset of cholangiocarcinomas arising from the gallbladder and hepatic ducts: continued caveats for utilization of immunohistochemistry panels. *Am J Surg Pathol.* (2014) 38:224–7. 10.1097/pas.0000000000000138 24418856

[40] Selves J Long-Mira E Mathieu M-C Rochaix P Ilié M. Immunohistochemistry for diagnosis of metastatic carcinomas of unknown primary site. *Cancers (Basel).* (2018) 10:108. 10.3390/cancers10040108 29621151 PMC5923363

[41] Miettinen M McCue PA Sarlomo-Rikala M Rys J Czapiewski P Wazny K GATA3: a multispecific but potentially useful marker in surgical pathology: a systematic analysis of 2500 epithelial and nonepithelial tumors. *Am J Surg Pathol.* (2014) 38:13–22. 10.1097/PAS.0b013e3182a0218f 24145643 PMC3991431

[42] Varma M Morgan M Jasani B Tamboli P Amin MB. Polyclonal anti-PSA is more sensitive but less specific than monoclonal anti-PSA: implications for diagnostic prostatic pathology. *Am J Clin Pathol.* (2002) 118:202–7. 10.1309/BGWQ-P26T-7TR6-VGT3 12162678

[43] Guedj N Zhan Q Perigny M Rautou PE Degos F Belghiti J Comparative protein expression profiles of hilar and peripheral hepatic cholangiocarcinomas. *J Hepatol.* (2009) 51:93–101. 10.1016/j.jhep.2009.03.017 19446907

[44] Shahid M Mubeen A Tse J Kakar S Bateman AC Borger D Branched chain in situ hybridization for albumin as a marker of hepatocellular differentiation: evaluation of manual and automated in situ hybridization platforms. *Am J Surg Pathol.* (2015) 39:25–34. 10.1097/PAS.0000000000000343 25353287 PMC4698370

[45] Ferrone CR Ting DT Shahid M Konstantinidis IT Sabbatino F Goyal L The ability to diagnose intrahepatic cholangiocarcinoma definitively using novel branched DNA-enhanced albumin RNA in situ hybridization technology. *Ann Surg Oncol.* (2016) 23:290–6. 10.1245/s10434-014-4247-8 25519926 PMC4472634

[46] Collins K Newcomb PH Cartun RW Ligato S. Utility and limitations of albumin mRNA in situ hybridization detection in the diagnosis of hepatobiliary lesions and metastatic carcinoma to the liver. *Appl Immunohistochem Mol Morphol.* (2021) 29:180–7. 10.1097/pai.0000000000000885 33208670

[47] Banales JM Huebert RC Karlsen T Strazzabosco M LaRusso NF Gores GJ. Cholangiocyte pathobiology. *Nat Rev Gastroenterol Hepatol.* (2019) 16:269–81. 10.1038/s41575-019-0125-y 30850822 PMC6563606

[48] Steiner PE Higginson J. Cholangiolocellular carcinoma of the liver. *Cancer.* (1959) 12:753–9. 10.1002/1097-0142(195907/08)12:4<753::aid-cncr2820120420>3.0.co;2-l 13663020

[49] Komuta M Spee B Vander Borght S De Vos R Verslype C Aerts R Clinicopathological study on cholangiolocellular carcinoma suggesting hepatic progenitor cell origin. *Hepatology.* (2008) 47:1544–56. 10.1002/hep.22238 18393293

[50] Moeini A Sia D Zhang Z Camprecios G Stueck A Dong H Mixed hepatocellular cholangiocarcinoma tumors: cholangiolocellular carcinoma is a distinct molecular entity. *J Hepatol.* (2017) 66:952–61. 10.1016/j.jhep.2017.01.010 28126467

[51] Liau JY Tsai JH Yuan RH Chang CN Lee HJ Jeng YM. Morphological subclassification of intrahepatic cholangiocarcinoma: etiological, clinicopathological, and molecular features. *Mod Pathol.* (2014) 27:1163–73. 10.1038/modpathol.2013.241 24406866

[52] Rhee H Ko JE Chung T Jee BA Kwon SM Nahm JH Transcriptomic and histopathological analysis of cholangiolocellular differentiation trait in intrahepatic cholangiocarcinoma. *Liver Int.* (2018) 38:113–24. 10.1111/liv.13492 28608943

[53] Sia D Hoshida Y Villanueva A Roayaie S Ferrer J Tabak B Integrative molecular analysis of intrahepatic cholangiocarcinoma reveals 2 classes that have different outcomes. *Gastroenterology.* (2013) 144:829–40. 10.1053/j.gastro.2013.01.001 23295441 PMC3624083

[54] Chung T Rhee H Shim HS Yoo JE Choi GH Kim H Genetic, clinicopathological, and radiological features of intrahepatic cholangiocarcinoma with ductal plate malformation pattern. *Gut Liver.* (2021). 10.5009/gnl210174 34810298 PMC9289835

[55] Nakanuma Y Sato Y Ikeda H Harada K Kobayashi M Sano K Intrahepatic cholangiocarcinoma with predominant “ductal plate malformation” pattern: a new subtype. *Am J Surg Pathol.* (2012) 36:1629–35. 10.1097/PAS.0b013e31826e0249 23073321

[56] Sasaki M Sato Y Nakanuma Y. Cholangiolocellular carcinoma with “ductal plate malformation” pattern may be characterized by ARID1A genetic alterations. *Am J Surg Pathol.* (2019) 43:352–60. 10.1097/pas.0000000000001201 30520820

[57] Gou Q Fu S Xie Y Zhang M Shen Y. Treatment and survival patterns of primary adenosquamous carcinoma of the liver: a retrospective analysis. *Front Oncol.* (2021) 11:621594. 10.3389/fonc.2021.621594 34434888 PMC8380844

[58] Nakanuma Y Sato Y Harada K Sasaki M Xu J Ikeda H. Pathological classification of intrahepatic cholangiocarcinoma based on a new concept. *World J Hepatol.* (2010) 2:419–27. 10.4254/wjh.v2.i12.419 21191517 PMC3010511

[59] Chi Z Bhalla A Saeed O Cheng L Curless K Wang HL Mucinous intrahepatic cholangiocarcinoma: a distinct variant. *Hum Pathol.* (2018) 78:131–7. 10.1016/j.humpath.2018.04.010 29698701

[60] Sumiyoshi T Shima Y Okabayashi T Ishikawa A Matsumoto M Iwata J Mucinous cholangiocarcinoma: clinicopathological features of the rarest type of cholangiocarcinoma. *Ann Gastroenterol Surg.* (2017) 1:114–21. 10.1002/ags3.12016 29863172 PMC5881371

[61] Haas S Gütgemann I Wolff M Fischer HP. Intrahepatic clear cell cholangiocarcinoma: immunohistochemical aspects in a very rare type of cholangiocarcinoma. *Am J Surg Pathol.* (2007) 31:902–6. 10.1097/PAS.0b013e31802c0c8a 17527078

[62] Yamamoto T Abe T Oshita A Yonehara S Katamura Y Matsumoto N Intrahepatic cholangiocarcinoma with clear cell type following laparoscopic curative surgery. *Surg Case Rep.* (2020) 6:264. 10.1186/s40792-020-01041-2 33026548 PMC7539241

[63] Guo XQ Li B Li Y Tian XY Li Z. Unusual mucoepidermoid carcinoma of the liver misdiagnosed as squamous cell carcinoma by intraoperative histological examination. *Diagn Pathol.* (2014) 9:24. 10.1186/1746-1596-9-24 24475740 PMC3906751

[64] Arakawa Y Shimada M Ikegami T Kubo T Imura S Morine Y Mucoepidermoid carcinoma of the liver: report of a rare case and review of the literature. *Hepatol Res.* (2008) 38:736–42. 10.1111/j.1872-034X.2008.00335.x 18336545

[65] Tsai J-H Liau J-Y Lee C-H Jeng Y-M. Lymphoepithelioma-like intrahepatic cholangiocarcinoma is a distinct entity with frequent pTERT/TP53 mutations and comprises 2 subgroups based on epstein-barr virus infection. *Am J Surg Pathol.* (2021) 45:1409–18. 10.1097/PAS.0000000000001716 33859071

[66] Khandakar B Liu J-R Thung S Li Y Rhee H Kagen AC Lymphoepithelioma-like neoplasm of the biliary tract with ‘probable low malignant potential’. *Histopathology.* (2022) 80:720–8. 10.1111/his.14580 34608670

[67] Malhotra S Wood J Mansy T Singh R Zaitoun A Madhusudan S. Intrahepatic sarcomatoid cholangiocarcinoma. *J Oncol.* (2010) 2010:701476. 10.1155/2010/701476 20454704 PMC2862318

[68] Matsukuma KE Yeh MM. Update on the pathology of liver neoplasms. *Ann Diagn Pathol.* (2019) 38:126–37. 10.1016/j.anndiagpath.2018.10.005 30597357

[69] Geramizadeh B. Precursor lesions of cholangiocarcinoma: a clinicopathologic review. *Clin Pathol.* (2020) 13:2632010X20925045. 10.1177/2632010x20925045 32596664 PMC7297471

[70] Who Classification of Tumours Editorial Board. *Digestive System Tumours.* 5th ed. Lyon: International Agency for Research on Cancer (2019).

[71] Nakanishi Y Zen Y Kondo S Itoh T Itatsu K Nakanuma Y. Expression of cell cycle-related molecules in biliary premalignant lesions: biliary intraepithelial neoplasia and biliary intraductal papillary neoplasm. *Hum Pathol.* (2008) 39:1153–61. 10.1016/j.humpath.2007.11.018 18495210

[72] Ettel M Eze O Xu R. Clinical and biological significance of precursor lesions of intrahepatic cholangiocarcinoma. *World J Hepatol.* (2015) 7:2563–70. 10.4254/wjh.v7.i25.2563 26557948 PMC4635141

[73] Hsu M Sasaki M Igarashi S Sato Y Nakanuma Y. KRAS and GNAS mutations and p53 overexpression in biliary intraepithelial neoplasia and intrahepatic cholangiocarcinomas. *Cancer.* (2013) 119:1669–74. 10.1002/cncr.27955 23335286

[74] Aishima S Fujita N Mano Y Kubo Y Tanaka Y Taketomi A Different roles of S100P overexpression in intrahepatic cholangiocarcinoma: carcinogenesis of perihilar type and aggressive behavior of peripheral type. *Am J Surg Pathol.* (2011) 35:590–8. 10.1097/PAS.0b013e31820ffdf1 21412073

[75] Nakanuma Y Kakuda Y Uesaka K Miyata T Yamamoto Y Fukumura Y Characterization of intraductal papillary neoplasm of bile duct with respect to histopathologic similarities to pancreatic intraductal papillary mucinous neoplasm. *Hum Pathol.* (2016) 51:103–13. 10.1016/j.humpath.2015.12.022 27067788

[76] Rocha FG Lee H Katabi N DeMatteo RP Fong Y D’Angelica MI Intraductal papillary neoplasm of the bile duct: a biliary equivalent to intraductal papillary mucinous neoplasm of the pancreas? *Hepatology.* (2012) 56:1352–60. 10.1002/hep.25786 22504729

[77] Kim KM Lee JK Shin JU Lee KH Lee KT Sung J-Y Clinicopathologic features of intraductal papillary neoplasm of the bile duct according to histologic subtype. *Am J Gastroenterol.* (2012) 107:118–25. 10.1038/ajg.2011.316 21946282

[78] Schlitter AM Born D Bettstetter M Specht K Kim-Fuchs C Riener M-O Intraductal papillary neoplasms of the bile duct: stepwise progression to carcinoma involves common molecular pathways. *Mod Pathol.* (2014) 27:73–86. 10.1038/modpathol.2013.112 23828315

[79] Nakanuma Y Uesaka K Kakuda Y Sugino T Kubota K Furukawa T Intraductal papillary neoplasm of bile duct: updated clinicopathological characteristics and molecular and genetic alterations. *J Clin Med.* (2020) 9:3991. 10.3390/jcm9123991 33317146 PMC7763595

[80] Nakanuma Y Uesaka K Okamura Y Terada T Fukumura Y Kakuda Y Reappraisal of pathological features of intraductal papillary neoplasm of bile duct with respect to the type 1 and 2 subclassifications. *Hum Pathol.* (2021) 111:21–35. 10.1016/j.humpath.2021.01.002 33508254

[81] Yang CY Huang WJ Tsai JH Cheng A Chen CC Hsu HP Targeted next-generation sequencing identifies distinct clinicopathologic and molecular entities of intraductal papillary neoplasms of the bile duct. *Mod Pathol.* (2019) 32:1637–45. 10.1038/s41379-019-0306-9 31231124

[82] Aoki Y Mizuma M Hata T Aoki T Omori Y Ono Y Intraductal papillary neoplasms of the bile duct consist of two distinct types specifically associated with clinicopathological features and molecular phenotypes. *J Pathol.* (2020) 251:38–48. 10.1002/path.5398 32100878

[83] Schlitter AM Jang K-T Klöppel G Saka B Hong S-M Choi H Intraductal tubulopapillary neoplasms of the bile ducts: clinicopathologic, immunohistochemical, and molecular analysis of 20 cases. *Mod Pathol.* (2015) 28:1249–64. 10.1038/modpathol.2015.61 26111977

[84] Goeppert B Stichel D Toth R Fritzsche S Loeffler MA Schlitter AM Integrative analysis reveals early and distinct genetic and epigenetic changes in intraductal papillary and tubulopapillary cholangiocarcinogenesis. *Gut.* (2022) 71:391–401. 10.1136/gutjnl-2020-322983 33468537 PMC8762040

[85] Fabris L Sato K Alpini G Strazzabosco M. The tumor microenvironment in cholangiocarcinoma progression. *Hepatology.* (2021) 73:75–85. 10.1002/hep.31410 32500550 PMC7714713

[86] Jing C-Y Fu Y-P Huang J-L Zhang M-X Yi Y Gan W Prognostic nomogram based on histological characteristics of fibrotic tumor stroma in patients who underwent curative resection for intrahepatic cholangiocarcinoma. *Oncologist.* (2018) 23:1482–93. 10.1634/theoncologist.2017-0439 30257891 PMC6292551

[87] Kajiyama K Maeda T Takenaka K Sugimachi K Tsuneyoshi M. The significance of stromal desmoplasia in intrahepatic cholangiocarcinoma: a special reference of ‘scirrhous-type’ and ‘nonscirrhous-type’. *Growth.* (1999) 23:892. 10.1097/00000478-199908000-00006 10435558

[88] Zhang X-F Dong M Pan Y-H Chen J-N Huang X-Q Jin Y Expression pattern of cancer-associated fibroblast and its clinical relevance in intrahepatic cholangiocarcinoma. *Hum Pathol.* (2017) 65:92–100. 10.1016/j.humpath.2017.04.014 28457731

[89] Kojima S Hisaka T Midorikawa R Naito Y Akiba J Tanigawa M Prognostic impact of desmoplastic reaction evaluation for intrahepatic cholangiocarcinoma. *Anticancer Res.* (2020) 40:4749–54. 10.21873/anticanres.14476 32727801

[90] De Jaeghere EA Denys HG De Wever O. Fibroblasts fuel immune escape in the tumor microenvironment. *Trends Cancer.* (2019) 5:704–23. 10.1016/j.trecan.2019.09.009 31735289

[91] Okabe H Beppu T Hayashi H Ishiko T Masuda T Otao R Hepatic stellate cells accelerate the malignant behavior of cholangiocarcinoma cells. *Ann Surg Oncol.* (2011) 18:1175–84. 10.1245/s10434-010-1391-7 21042948

[92] Clapéron A Mergey M Aoudjehane L Ho-Bouldoires THN Wendum D Prignon A Hepatic myofibroblasts promote the progression of human cholangiocarcinoma through activation of epidermal growth factor receptor. *Hepatology.* (2013) 58:2001–11. 10.1002/hep.26585 23787814

[93] Fingas CD Bronk SF Werneburg NW Mott JL Guicciardi ME Cazanave SC Myofibroblast-derived PDGF-BB promotes hedgehog survival signaling in cholangiocarcinoma cells. *Hepatology.* (2011) 54:2076–88. 10.1002/hep.24588 22038837 PMC3230714

[94] Chuaysri C Thuwajit P Paupairoj A Chau-In S Suthiphongchai T Thuwajit C. Alpha-smooth muscle actin-positive fibroblasts promote biliary cell proliferation and correlate with poor survival in cholangiocarcinoma. *Oncol Rep.* (2009) 21:957–69. 10.3892/or_00000309 19287994

[95] Shan Z Ju C. Hepatic macrophages in liver injury. *Front Immunol.* (2020) 11:322. 10.3389/fimmu.2020.00322 32362892 PMC7180226

[96] Sato K Hall C Glaser S Francis H Meng F Alpini G. Pathogenesis of Kupffer cells in cholestatic liver injury. *Am J Pathol.* (2016) 186:2238–47. 10.1016/j.ajpath.2016.06.003 27452297 PMC5012503

[97] Yuan H Lin Z Liu Y Jiang Y Liu K Tu M Intrahepatic cholangiocarcinoma induced M2-polarized tumor-associated macrophages facilitate tumor growth and invasiveness. *Cancer Cell Int.* (2020) 20:586. 10.1186/s12935-020-01687-w 33372604 PMC7720384

[98] Høgdall D Lewinska M Andersen JB. Desmoplastic tumor microenvironment and immunotherapy in cholangiocarcinoma. *Trends Cancer.* (2018) 4:239–55. 10.1016/j.trecan.2018.01.007 29506673

[99] Hasita H Komohara Y Okabe H Masuda T Ohnishi K Lei XF Significance of alternatively activated macrophages in patients with intrahepatic cholangiocarcinoma. *Cancer Sci.* (2010) 101:1913–9. 10.1111/j.1349-7006.2010.01614.x 20545696 PMC11158749

[100] Kasper HU Drebber U Stippel DL Dienes HP Gillessen A. Liver tumor infiltrating lymphocytes: comparison of hepatocellular and cholangiolar carcinoma. *World J Gastroenterol.* (2009) 15:5053–7. 10.3748/wjg.15.5053 19859998 PMC2768884

[101] Goeppert B Frauenschuh L Zucknick M Stenzinger A Andrulis M Klauschen F Prognostic impact of tumour-infiltrating immune cells on biliary tract cancer. *Br J Cancer.* (2013) 109:2665–74. 10.1038/bjc.2013.610 24136146 PMC3833207

[102] Yugawa K Itoh S Yoshizumi T Iseda N Tomiyama T Toshima T Prognostic impact of tumor microvessels in intrahepatic cholangiocarcinoma: association with tumor-infiltrating lymphocytes. *Mod Pathol.* (2021) 34:798–807. 10.1038/s41379-020-00702-9 33077921

[103] Kim H-D Kim JH Ryu Y-M Kim D Lee S Shin J Spatial distribution and prognostic implications of tumor-infiltrating FoxP3- CD4+ T cells in biliary tract cancer. *Cancer Res Treat.* (2021) 53:162–71. 10.4143/crt.2020.704 32878426 PMC7812013

[104] Ghidini M Cascione L Carotenuto P Lampis A Trevisani F Previdi MC Characterisation of the immune-related transcriptome in resected biliary tract cancers. *Eur J Cancer.* (2017) 86:158–65. 10.1016/j.ejca.2017.09.005 28988016 PMC5699791

[105] Fontugne J Augustin J Pujals A Compagnon P Rousseau B Luciani A PD-L1 expression in perihilar and intrahepatic cholangiocarcinoma. *Oncotarget.* (2017) 8:24644–51. 10.18632/oncotarget.15602 28445951 PMC5421876

[106] Wu H Wei Y Jian M Lu H Song Q Hao L Clinicopathological and prognostic significance of immunoscore and PD-L1 in intrahepatic cholangiocarcinoma. *OncoTargets Ther.* (2021) 14:39–51. 10.2147/ott.S288982 33442265 PMC7797318

[107] Sabbatino F Villani V Yearley JH Deshpande V Cai L Konstantinidis IT PD-L1 and HLA class I antigen expression and clinical course of the disease in intrahepatic cholangiocarcinoma. *J Clin Cancer Res.* (2016) 22:470–8. 10.1158/1078-0432.CCR-15-0715 26373575 PMC5296951

[108] Rizzo A Ricci AD Brandi G. PD-L1, TMB, MSI, and other predictors of response to immune checkpoint inhibitors in biliary tract cancer. *Cancers (Basel).* (2021) 13:558. 10.3390/cancers13030558 33535621 PMC7867133

[109] Wardell CP Fujita M Yamada T Simbolo M Fassan M Karlic R Genomic characterization of biliary tract cancers identifies driver genes and predisposing mutations. *J Hepatol.* (2018) 68:959–69. 10.1016/j.jhep.2018.01.009 29360550

[110] Terashima T Umemoto K Takahashi H Hosoi H Takai E Kondo S Germline mutations in cancer-predisposition genes in patients with biliary tract cancer. *Oncotarget.* (2019) 10:5949–57. 10.18632/oncotarget.27224 31666926 PMC6800267

[111] Maynard H Stadler ZK Berger MF Solit DB Ly M Lowery MA Germline alterations in patients with biliary tract cancers: a spectrum of significant and previously underappreciated findings. *Cancer.* (2020) 126:1995–2002. 10.1002/cncr.32740 32012241 PMC7584349

[112] Golan T Raitses-Gurevich M Kelley RK Bocobo AG Borgida A Shroff RT Overall survival and clinical characteristics of BRCA-associated cholangiocarcinoma: a multicenter retrospective study. *Oncologist.* (2017) 22:804–10. 10.1634/theoncologist.2016-0415 28487467 PMC5507643

[113] Brandi G Deserti M Palloni A Turchetti D Zuntini R Pedica F Intrahepatic cholangiocarcinoma development in a patient with a novel BAP1 germline mutation and low exposure to asbestos. *Cancer Genet.* (2020) 24:57–62. 10.1016/j.cancergen.2020.10.001 33093002

[114] Kiba T Tsuda H Hirohashi S Inoue S Sugimura T Pairojkul C. Mutations of the p53 tumor suppressor gene and the ras gene family in intrahepatic cholangiocellular carcinomas in Japan and Thailand. *Mol Carcinog.* (1993) 8:312–8. 10.1002/mc.2940080415 8280380

[115] Ross JS Wang K Gay L Al-Rohil R Rand JV Jones DM New routes to targeted therapy of intrahepatic cholangiocarcinomas revealed by next-generation sequencing. *Oncologist.* (2014) 19:235–42. 10.1634/theoncologist.2013-0352 24563076 PMC3958461

[116] Chaisaingmongkol J Budhu A Dang H Rabibhadana S Pupacdi B Kwon SM Common molecular subtypes among asian hepatocellular carcinoma and cholangiocarcinoma. *Cancer Cell.* (2017) 32:57–70.e3. 10.1016/j.ccell.2017.05.009 28648284 PMC5524207

[117] Simbolo M Fassan M Ruzzenente A Mafficini A Wood LD Corbo V Multigene mutational profiling of cholangiocarcinomas identifies actionable molecular subgroups. *Oncotarget.* (2014) 5:2839–52. 10.18632/oncotarget.1943 24867389 PMC4058049

[118] Churi CR Shroff R Wang Y Rashid A Kang HC Weatherly J Mutation profiling in cholangiocarcinoma: prognostic and therapeutic implications. *PLoS One.* (2014) 9:e115383. 10.1371/journal.pone.0115383 25536104 PMC4275227

[119] Jiao Y Pawlik TM Anders RA Selaru FM Streppel MM Lucas DJ Exome sequencing identifies frequent inactivating mutations in BAP1, ARID1A and PBRM1 in intrahepatic cholangiocarcinomas. *Nat Genet.* (2013) 45:1470–3. 10.1038/ng.2813 24185509 PMC4013720

[120] Nakamura H Arai Y Totoki Y Shirota T Elzawahry A Kato M Genomic spectra of biliary tract cancer. *Nat Genet.* (2015) 47:1003–10. 10.1038/ng.3375 26258846

[121] Fujimoto A Furuta M Shiraishi Y Gotoh K Kawakami Y Arihiro K Whole-genome mutational landscape of liver cancers displaying biliary phenotype reveals hepatitis impact and molecular diversity. *Nat Commun.* (2015) 6:6120. 10.1038/ncomms7120 25636086

[122] Wang T Drill E Vakiani E Pak LM Boerner T Askan G Distinct histomorphological features are associated with IDH1 mutation in intrahepatic cholangiocarcinoma. *Hum Pathol.* (2019) 91:19–25. 10.1016/j.humpath.2019.05.002 31121195 PMC6744317

[123] Jolissaint JS Soares KC Seier KP Kundra R Gönen M Shin PJ Intrahepatic cholangiocarcinoma with lymph node metastasis: treatment-related outcomes and the role of tumor genomics in patient selection. *Clin Cancer Res.* (2021) 27:4101–8. 10.1158/1078-0432.CCR-21-0412 33963001 PMC8282702

[124] Kipp BR Voss JS Kerr SE Barr Fritcher EG Graham RP Zhang L Isocitrate dehydrogenase 1 and 2 mutations in cholangiocarcinoma. *Hum Pathol.* (2012) 43:1552–8. 10.1016/j.humpath.2011.12.007 22503487

[125] Zhu AX Borger DR Kim Y Cosgrove D Ejaz A Alexandrescu S Genomic profiling of intrahepatic cholangiocarcinoma: refining prognosis and identifying therapeutic targets. *Ann Surg Oncol.* (2014) 21:3827–34. 10.1245/s10434-014-3828-x 24889489 PMC4324507

[126] Boerner T Drill E Pak LM Nguyen B Sigel CS Doussot A Genetic determinants of outcome in intrahepatic cholangiocarcinoma. *Hepatology.* (2021) 74:1429–44. 10.1002/hep.31829 33765338 PMC8713028

[127] Nepal C O’Rourke CJ Oliveira DVNP Taranta A Shema S Gautam P Genomic perturbations reveal distinct regulatory networks in intrahepatic cholangiocarcinoma. *Hepatology.* (2018) 68:949–63. 10.1002/hep.29764 29278425 PMC6599967

[128] Lowery MA Ptashkin R Jordan E Berger MF Zehir A Capanu M Comprehensive molecular profiling of intrahepatic and extrahepatic cholangiocarcinomas: potential targets for intervention. *Clin Cancer Res.* (2018) 24:4154–61. 10.1158/1078-0432.CCR-18-0078 29848569 PMC6642361

[129] Borad MJ Champion MD Egan JB Liang WS Fonseca R Bryce AH Integrated genomic characterization reveals novel, therapeutically relevant drug targets in FGFR and EGFR pathways in sporadic intrahepatic cholangiocarcinoma. *PLoS Genet.* (2014) 10:e1004135. 10.1371/journal.pgen.1004135 24550739 PMC3923676

[130] Goyal L Kongpetch S Crolley VE Bridgewater J. Targeting FGFR inhibition in cholangiocarcinoma. *Cancer Treat Rev.* (2021) 95:102170. 10.1016/j.ctrv.2021.102170 33735689

[131] Jusakul A Cutcutache I Yong CH Lim JQ Huang MN Padmanabhan N Whole-genome and epigenomic landscapes of etiologically distinct subtypes of cholangiocarcinoma. *Cancer Discov.* (2017) 7:1116–35. 10.1158/2159-8290.CD-17-0368 28667006 PMC5628134

[132] Winkelmann R Schneider M Hartmann S Schnitzbauer AA Zeuzem S Peveling-Oberhag J Microsatellite instability occurs rarely in patients with cholangiocarcinoma: a retrospective study from a German tertiary care hospital. *Int J Mol Sci.* (2018) 19:1421. 10.3390/ijms19051421 29747443 PMC5983652

[133] Goeppert B Roessler S Renner M Singer S Mehrabi A Vogel MN Mismatch repair deficiency is a rare but putative therapeutically relevant finding in non-liver fluke associated cholangiocarcinoma. *Br J Cancer.* (2019) 120:109–14. 10.1038/s41416-018-0199-2 30377340 PMC6325153

[134] Li K Luo H Huang L Luo H Zhu X. Microsatellite instability: a review of what the oncologist should know. *Cancer Cell Int.* (2020) 20:16. 10.1186/s12935-019-1091-8 31956294 PMC6958913

[135] Ohashi K Nakajima Y Kanehiro H Tsutsumi M Taki J Aomatsu Y Ki-ras mutations and p53 protein expressions in intrahepatic cholangiocarcinomas: relation to gross tumor morphology. *Gastroenterology.* (1995) 109:1612–7. 10.1016/0016-5085(95)90650-9 7557145

[136] Goeppert B Toth R Singer S Albrecht T Lipka DB Lutsik P Integrative analysis defines distinct prognostic subgroups of intrahepatic cholangiocarcinoma. *Hepatology.* (2019) 69:2091–106. 10.1002/hep.30493 30615206 PMC6594081

[137] Ma B Meng H Tian Y Wang Y Song T Zhang T Distinct clinical and prognostic implication of IDH1/2 mutation and other most frequent mutations in large duct and small duct subtypes of intrahepatic cholangiocarcinoma. *BMC Cancer.* (2020) 20:318. 10.1186/s12885-020-06804-6 32293336 PMC7161164

[138] Chan-on W Nairismägi M-L Ong CK Lim WK Dima S Pairojkul C Exome sequencing identifies distinct mutational patterns in liver fluke–related and non-infection-related bile duct cancers. *Nat Genet.* (2013) 45:1474–8. 10.1038/ng.2806 24185513

[139] Zou S Li J Zhou H Frech C Jiang X Chu JSC Mutational landscape of intrahepatic cholangiocarcinoma. *Nat Commun.* (2014) 5:5696. 10.1038/ncomms6696 25526346

[140] Andersen JB Spee B Blechacz BR Avital I Komuta M Barbour A Genomic and genetic characterization of cholangiocarcinoma identifies therapeutic targets for tyrosine kinase inhibitors. *Gastroenterology.* (2012) 142:1021–31.e15. 10.1053/j.gastro.2011.12.005 22178589 PMC3413201

[141] Ashburner M Ball CA Blake JA Botstein D Butler H Cherry JM Gene ontology: tool for the unification of biology. *Nat Genet.* (2000) 25:25–9. 10.1038/75556 10802651 PMC3037419

[142] Job S Rapoud D Dos Santos A Gonzalez P Desterke C Pascal G Identification of four immune subtypes characterized by distinct composition and functions of tumor microenvironment in intrahepatic cholangiocarcinoma. *Hepatology.* (2020) 72:965–81. 10.1002/hep.31092 31875970 PMC7589418

[143] Rizzo A Ricci AD Brandi G. IDH inhibitors in advanced cholangiocarcinoma: another arrow in the quiver? *Cancer Treat Res Commun.* (2021) 27:100356. 10.1016/j.ctarc.2021.100356 33799004

[144] Zhu AX Macarulla T Javle MM Kelley RK Lubner SJ Adeva J Final overall survival efficacy results of Ivosidenib for patients with advanced cholangiocarcinoma with IDH1 mutation: the phase 3 randomized clinical ClarIDHy trial. *JAMA Oncol.* (2021) 7:1669–77. 10.1001/jamaoncol.2021.3836 34554208 PMC8461552

[145] Acher AW Paro A Elfadaly A Tsilimigras D Pawlik TM. Intrahepatic cholangiocarcinoma: a summative review of biomarkers and targeted therapies. *Cancers (Basel).* (2021) 13:5169. 10.3390/cancers13205169 34680318 PMC8533913

[146] Mondaca S Razavi P Xu C Offin M Myers M Scaltriti M Genomic characterization of ERBB2-driven biliary cancer and a case of response to Ado-Trastuzumab Emtansine. *JCO Precis Oncol.* (2019) 3:1–9. 10.1200/po.19.00223 32923849 PMC7446346

[147] Mosele F Remon J Mateo J Westphalen CB Barlesi F Lolkema MP Recommendations for the use of next-generation sequencing (NGS) for patients with metastatic cancers: a report from the ESMO precision medicine working group. *Ann Oncol.* (2020) 31:1491–505. 10.1016/j.annonc.2020.07.014 32853681

